# Supplementary material for: Membrane lipid poly-unsaturation selectively affects dopamine D2 receptor endocytosis
Source: Nat Commun. 2026 May 20;17:6661. doi: 10.1038/s41467-026-73057-5 (PMC13381900; doi:10.1038/s41467-026-73057-5)
Supplement: Supplementary file 1 — Supplementary Information [file 41467_2026_73057_MOESM1_ESM.pdf]

Supplementary Figures and Tables

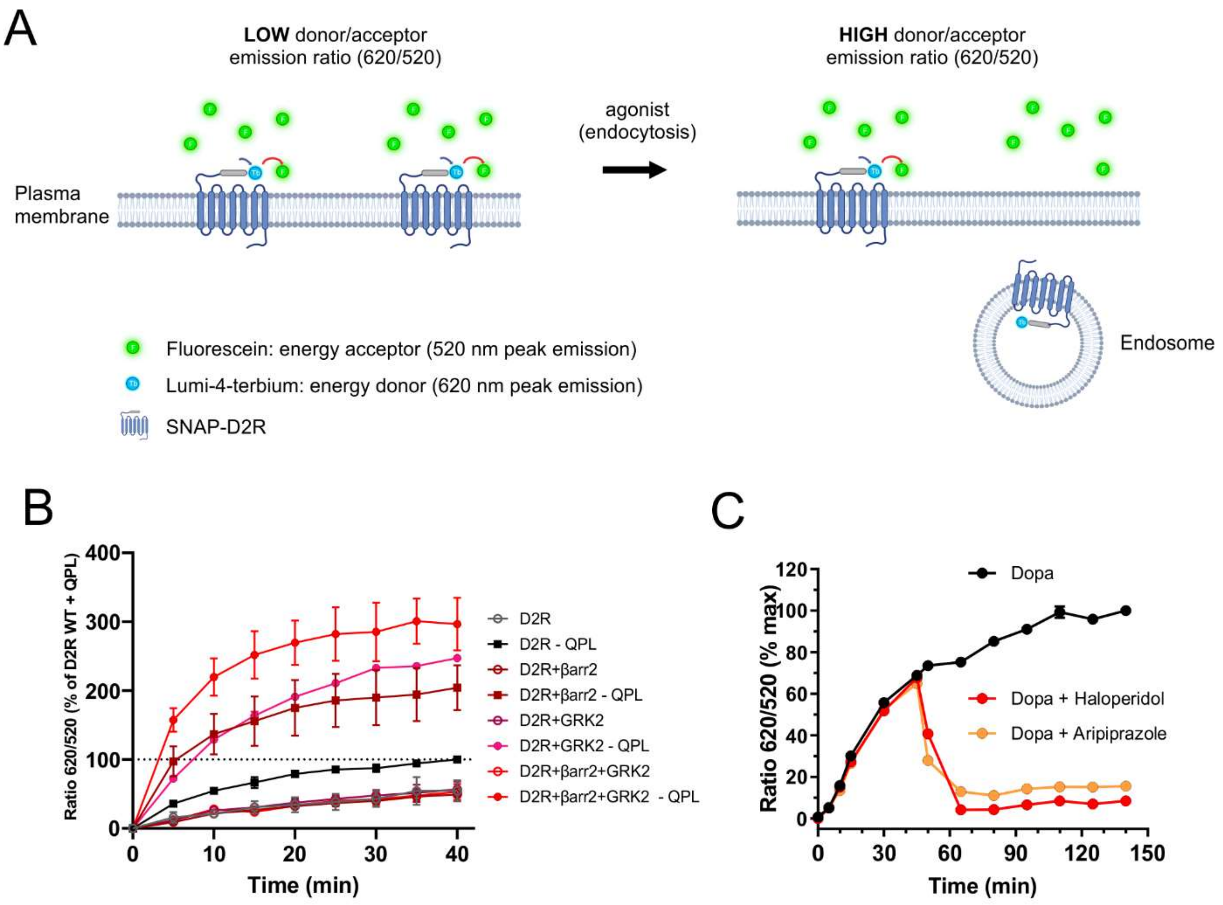

Supplementary Fig. 1: Validation of the DERET assay to measure D2R internalization after agonist stimulation. **A**, Principle of DERET assay. In absence of its agonist, the D2R covalently labeled with cell-impermeable SNAP-Lumi4®-Tb (energy donor, blue) is at the cell surface. Addition of excess of fluorescein (energy acceptor, green dots) in the extracellular medium leads to efficient energy transfer (red arrows) resulting in a low DERET ratio (620/520 nm). Following agonist addition, D2R is endocytosed which causes a significant reduction of energy transfer to the acceptor resulting in higher DERET ratio. Created in BioRender. Sposini, S. (2026) https://BioRender.com/iggmi1b **B**, Real-time internalization of SNAP-D2R following application of QPL (10 µM) (filled squares) or not (hollow circles) at time 0 in cells transfected with SNAP-D2R alone (black) or in combination with β-arrestin2 (brown), GRK2 (pink), or both (red). Circles and error bars represent mean ± SEM for 3 independent experiments. **C**, Real-time internalization of SNAP-D2R following Dopamine (Dopa) addition alone (10 µM, at t0) or upon addition at t = 50 min of the antagonist Haloperidol (100 µM) or the partial agonist Aripiprazole (100 µM). Circles represent mean of 2 independent experiments.In B and C, Percentages of fluorescence ratio R (620/520 nm) are plotted as a function of time.

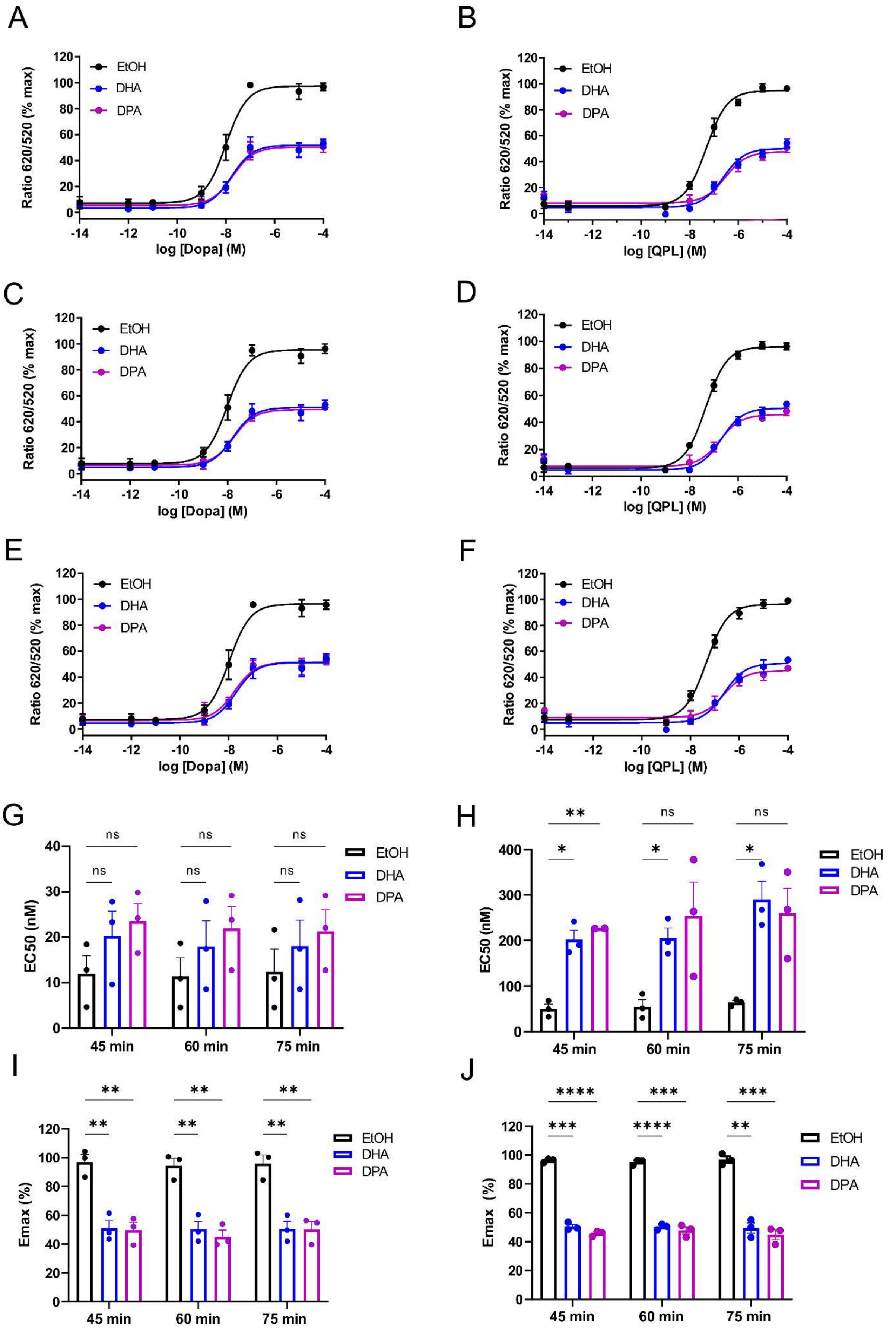

Supplementary Fig.2: Impact of PUFAs on D2R induced internalization investigated by DERET assay A-F: Dose–response curves of D2R induced internalization. Control and

PUFA-enriched (DHA and DPA) HEK cells expressing SNAP-D2R were incubated in the presence of an increased concentration of either Dopamine (Dopa) or Quinpirole (QPL) for 45 min (A, B), 60 min (C, D), 75 min (E, F) at 37°C. Data were fitted using non-linear regression dose–response log [ligand] versus response with three parameters. **G**, **H**: EC50 values calculated from dose-response curves obtained at later time points (45, 60, 75 min) following stimulation with either Dopamine (G) or Quinpirole (H). **I**, **J**: Emax values measured from dose-response curves obtained at later time points (45, 60, 75 min) following stimulation with either Dopamine (I) or Quinpirole (J). For all panels, mean ± SD of 3 independent experiments. Two-way ANOVA with Dunnett's multiple comparisons test of n=3 independent experiments carried out in triplicates; **** $p < 0.0001$, ** $p < 0.01$, * $p < 0.05$, ns $p \geq 0.05$.

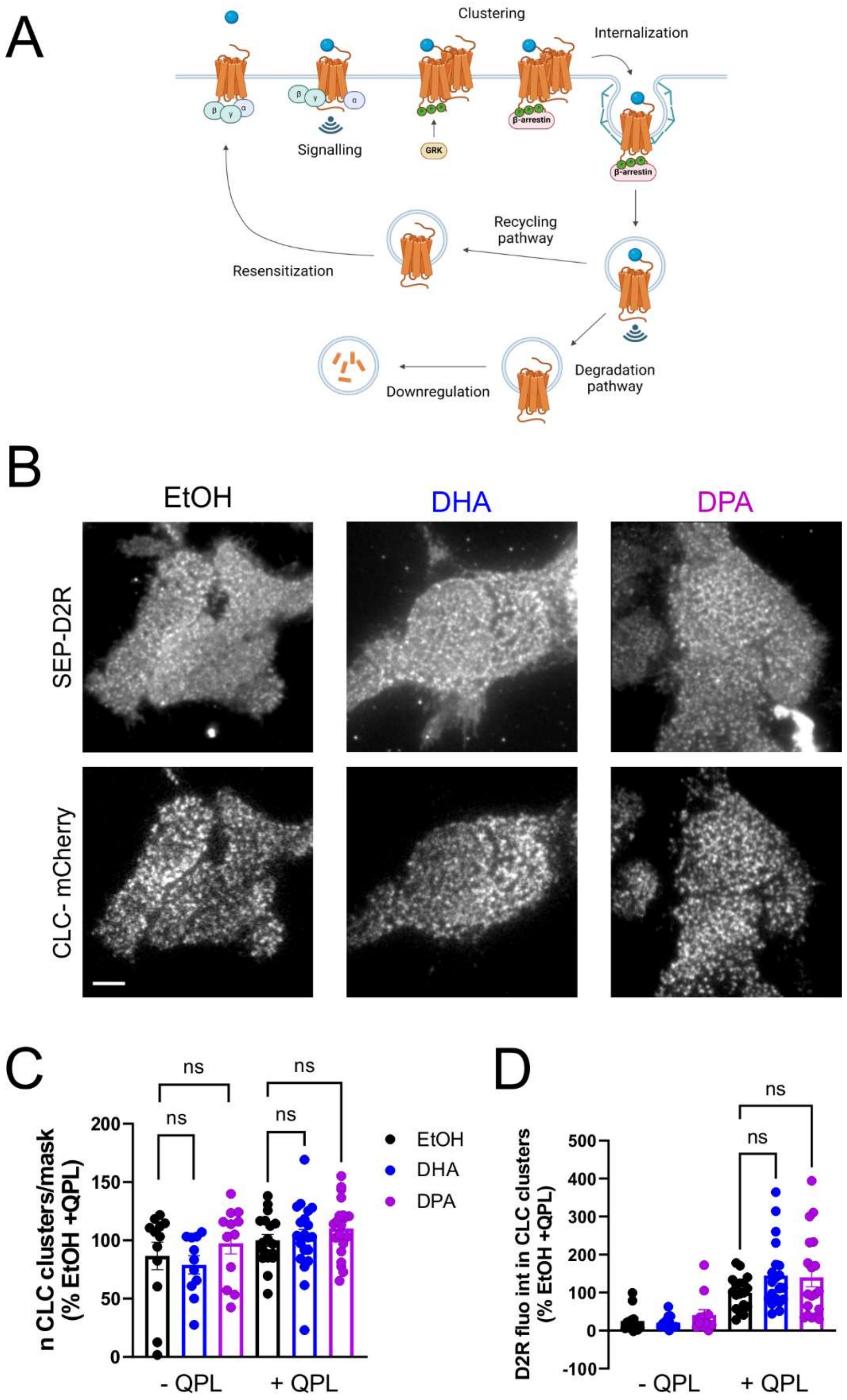

Supplementary Fig.3: PUFA treatments do not affect clustering of D2R to CCPs. A, Scheme of the key steps and proteins involved in D2R internalization, which takes place at CCPs. Created in BioRender. Sposini, S. (2026) https://BioRender.com/c0sxfz6. **B**, Representative images of HEK-293 cells transfected with SEP-D2R, CLC-mCherry,β-arr2 and GRK2, enriched in either ethanol (EtOH), DHA or DPA, treated with QPL (10 μM) for 10 minutes and imaged live by TIRF microscopy. Scale bar = 5 μm. **C**, Quantification of the number of CCPs per cell, from cells as in B n = 12-20 cells/condition collected across 3 independent experiments. One-way ANOVA followed by Dunnett's multiple comparison test. ns $p>0.05$. **D**, Quantification of D2R fluorescence in CCPs relative to D2R fluorescence outside CCPs, before (-QPL) and after stimulation with QPL (+QPL), from cells as in B

n=12-20 cells/condition collected across 3 independent experiments. One-way ANOVA followed by Dunnett's multiple comparison test. ns $p \geq 0.05$.

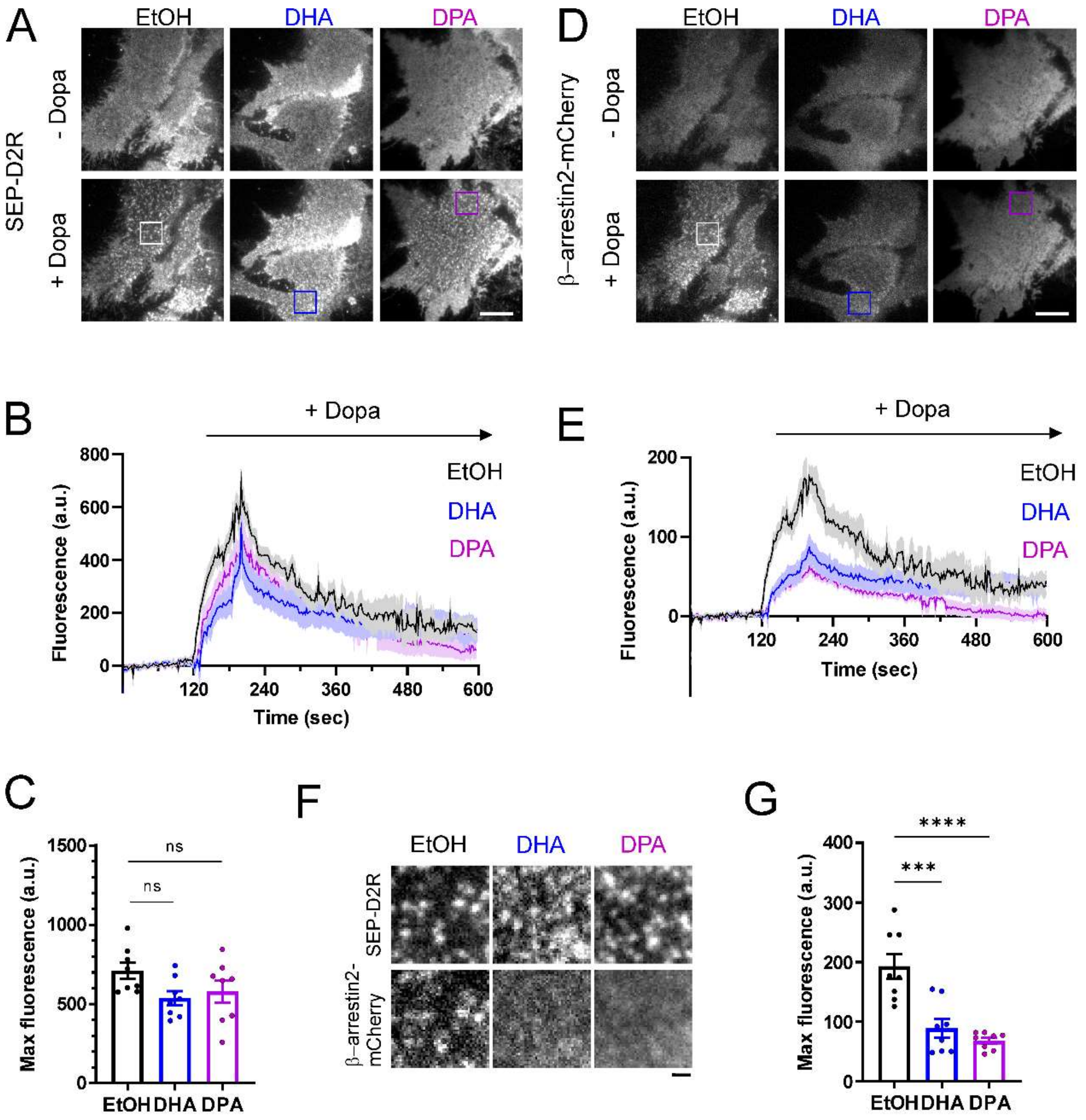

Supplementary Fig.4: PUFA enrichment does not affect D2R clustering but impairs the recruitment of β-arr2 upon Dopamine addition. A, D: Representative TIRF microscopy images of HEK293 cells co-expressing SEP-D2R, GRK2 and β-arrestin2-mCherry enriched in either ethanol (EtOH), DHA or DPA, showing the pattern of expression at the plasma membrane for SEP-D2R (A) and β-arrestin2-mCherry (D) before and after stimulation with 10 µM of Dopamine (Dopa). Scale bar 5 µm. **B, E:** Mean Fluorescence intensity profiles of SEP-D2R (B) and β-arrestin 2-mCherry (E) obtained before and after Dopamine (Dopa) addition at t=120 s. Data represent average fluorescence values within an ROI drawn around each cluster minus background fluorescence (= fluorescence measured in the ROI during the 60 frames before agonist addition was averaged and subtracted from fluorescence values at each frame). **C, G:** Maximum fluorescence intensities of SEP-D2R (C) and β-arrestin2-mCherry (G) measured after Dopamine addition. Values represent maximum intensity fluorescence minus background fluorescence in each ROI for n= 8 cells per condition collected across 2 independent experiments. One-way ANOVA test with Dunnett's multiple comparisons test; *** $p < 0.005$, ns $p \geq 0.05$. **F:** Zoom-in images taken from panels in A and D, as shown by ROIs, depicting SEP-D2R and β-arrestin2-mCherry clusters after Dopamine addition. Scale bar= 1 µm.

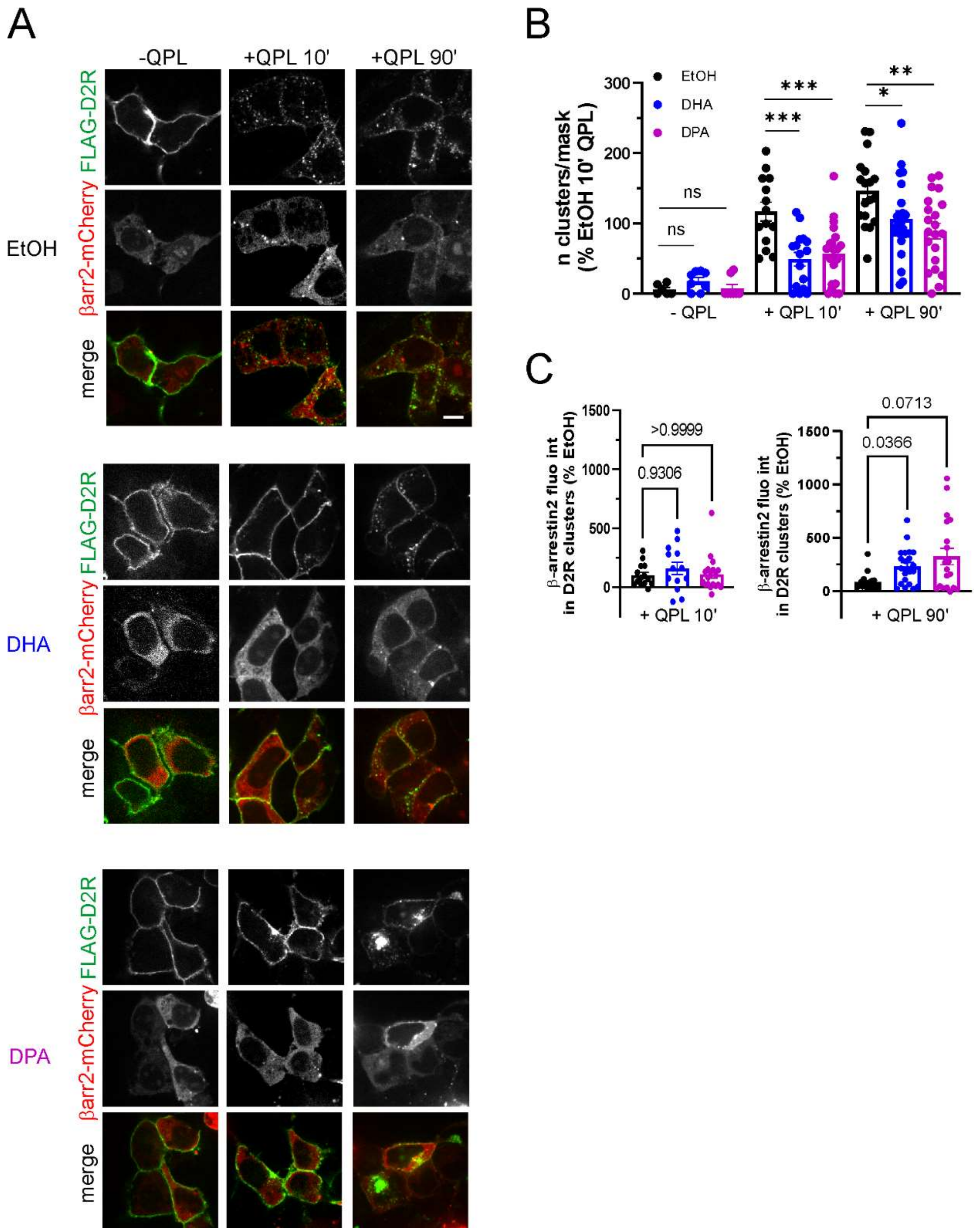

Supplementary Fig. 5: DHA treatment increases recruitment of βarr2 to D2R intracellular clusters at 90 minutes of QPL stimulation. A, Representative confocal microscopy images of HEK293 cells co-expressing FLAG-D2R, GRK2 and βarrestin2-mCherry enriched in either ethanol (EtOH), DHA or DPA, and incubated with anti-FLAG M1 antibody and stimulated without QPL (- QPL) or with QPL for 10 (+ QPL 10') or 90 minutes (+ QPL 90'). **B**, Quantification of the number of intracellular FLAG-D2R clusters per cell in all 9 conditions from cells as in A. n = 6-22 cells/condition collected across 2 independent experiments. One-way ANOVA followed by Dunnett's multiple comparison test: *** $p<0.001$, ** $p<0.01$, * $p<0.05$, ns $p\geq 0.05$. **C**, Quantification of βarrestin2-mCherry fluorescence in D2R clusters relative to βarrestin2-mCherry fluorescence outside D2R clusters, after stimulation with QPL for 10 (+ QPL 10') or 90 minutes (+ QPL 90'), from cells

as in A. n= 6-22 cells/condition collected across 2 independent experiments. Kruskal-Wallis test followed by Dunn's multiple comparisons test: * p<0.05, ns >0.05.

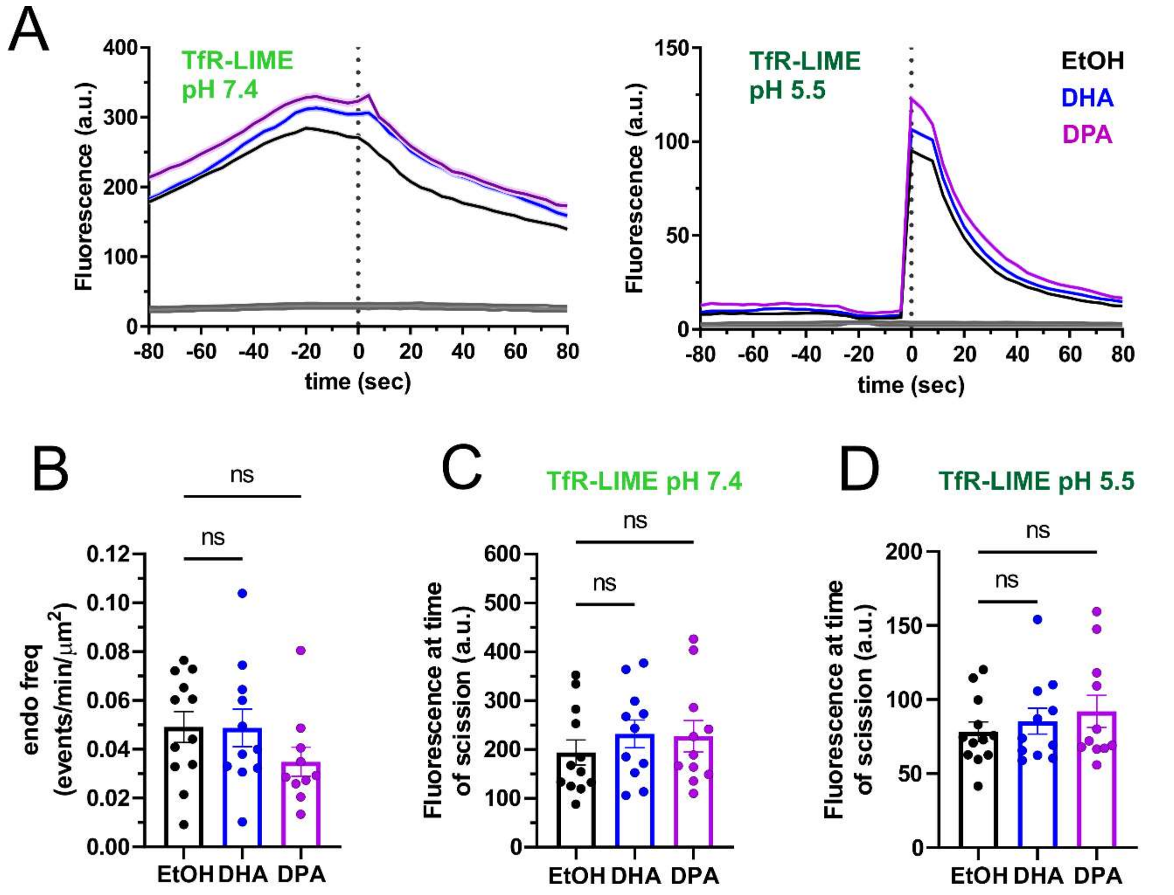

Supplementary Fig. 6: PUFA enrichment does not affect TfR-SEP endocytosis measured with the ppH assay. A: Average fluorescence over time of TfR-Lime at pH 7.4 and TfR-LIME at pH 5.5 aligned to the time of vesicle scission (t = 0 s) obtained from terminal events (events for which the TfR-Lime cluster at pH 7.4 disappears within 80 s, defined as in (Taylor *et al*, 2011; Sposini *et al*, 2020) from HEK293 cells transfected with TfR-Lime, treated with either EtOH, DHA or DPA and imaged live with the ppH protocol. **B:** Number of endocytic events/min/µm^2 detected in the same cells as in A. One-way ANOVA with Dunnett's multiple comparisons test of 12, 11 and 10 cells treated with EtOH, DHA or DPA, respectively; ns p ≤ 0.05. **C-D:** Average fluorescence intensity of all events in a given cell, at the time of endocytic event detection, for TfR-Lime at pH 7.4 (C) and TfR-Lime at pH 5.5 obtained from the same cells as in A. One-way ANOVA with Dunnett's multiple comparisons test of 12, 11 and 10 cells treated with EtOH, DHA or DPA, respectively; ns p ≤ 0.05.

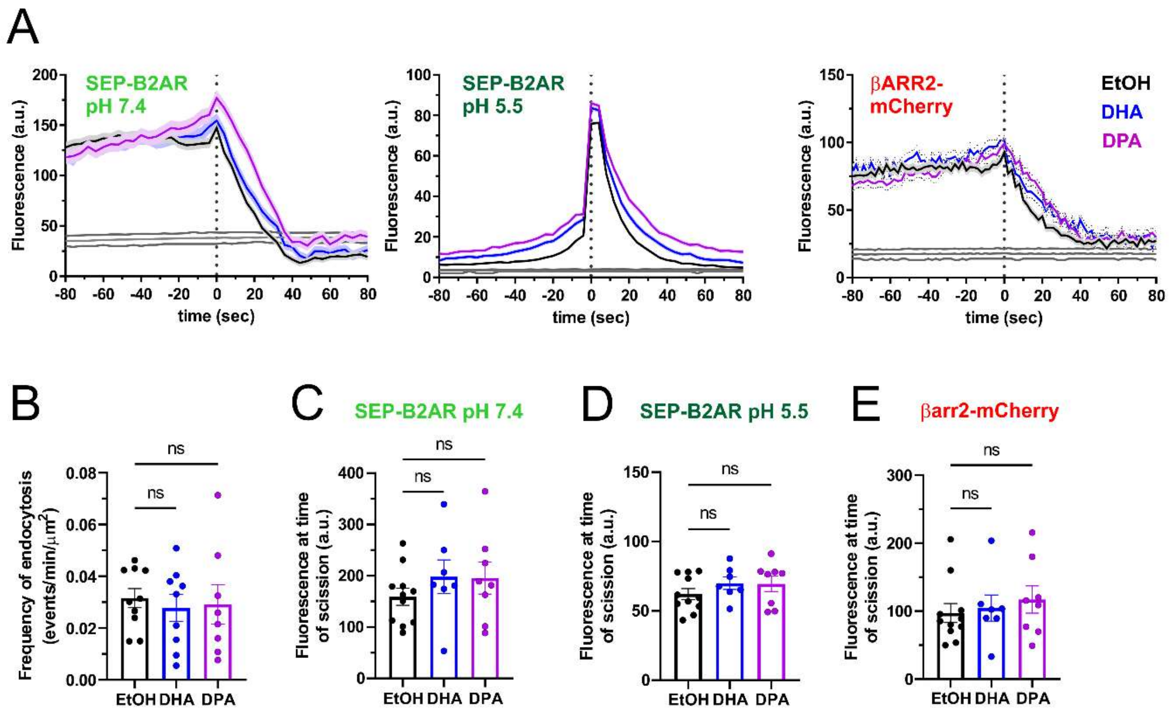

Supplementary Fig. 7: PUFA enrichment does not affect SEP-β2AR endocytosis induced by Iso and measured with the ppH assay. A: Average fluorescence over time of SEP-B2AR at pH 7.4, SEP-B2AR at pH 5.5 and β-arrestin2-mCherry at pH 5.5, aligned to the time of vesicle scission (t = 0 s) obtained from terminal events (events for which the SEP-B2AR cluster at pH 7.4 disappears within 80 s, defined as in (Taylor *et al*, 2011; Sposini *et al*, 2020) from HEK293 cells transfected with SEP-B2AR, βarr2-mCherry and GRK2, treated with either EtOH, DHA or DPA and imaged live with the ppH protocol before (0-120 s), during (121-720 s) and after (701-900 s) application of 100 nM Isoproterenol. **B:** Number of endocytic events/min/μm^2 detected during Isoproterenol application in the same cells as in A. One-way ANOVA with Dunnett's multiple comparisons test of 10, 9 and 8 cells treated with EtOH, DHA or DPA, respectively; ns $p \leq 0.05$. **C-E:** Average fluorescence intensity of all events in a given cell, at the time of endocytic event detection, for SEP-D2R at pH7.4 (C), SEP-D2R at pH 5.5 (D) and β-arrestin2-mCherry at pH 5.5 (D) obtained from the same cells as in A. One-way ANOVA with Dunnett's multiple comparisons test of 10, 9 and 8 cells treated with EtOH, DHA or DPA, respectively; ns $p \leq 0.05$.

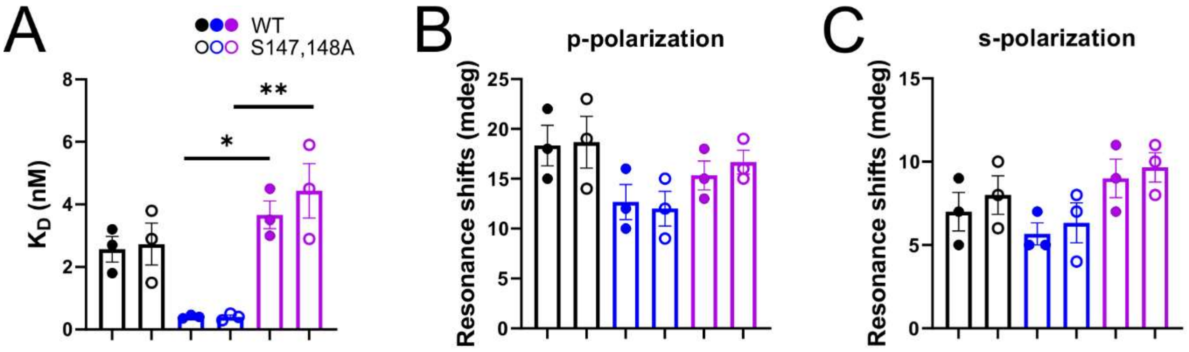

Supplementary Fig.8: The modulation of D2R affinity for quinpirole by DHA and DPA is the same for WT and S147, 148A mutant receptors. A, QPL binding affinity to D2R WT (filled circles) and S147,148A mutant in membranes from cells treated with EtOH (black), DHA (blue) or DPA (magenta). One-way ANOVA with Dunnett's multiple comparisons test of WT DHA vs DPA $p = 0.0107$; or mut DHA vs DPA $p = 0.0018$. **B-C**, conformational changes induced by QPL in PUFA-enriched cell membranes observed by PWR with p-polarization (B) and s-polarization (C). Membranes from non-treated cells are presented in black, DHA- and DPA-enriched in blue and magenta, respectively. All panels, Mean ± SD of 3 independent experiments.

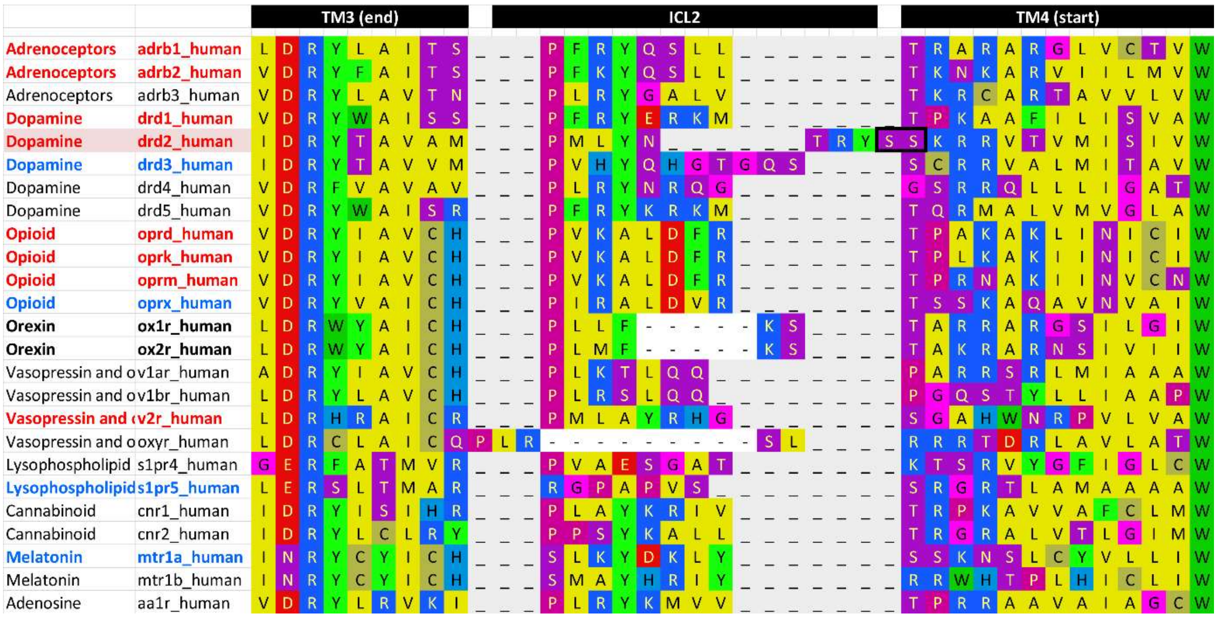

Supplementary Fig. 9: Alignment of residues in regions TM3, ICL2 and TM4 of a selection of class A GPCRs. Alignment performed with GPCRdb. Left, agonists and names of human GPCRs (UniProt). In red, the ones tested in this study for their dependency to DHA and DPA enrichment. In blue, receptors containing consecutive serines at the junction between ICL2 and TM4. Black rectangle highlights consecutive serine residues (S) at the end of ICL2 of D2R (S147 and S148). Residues (in one letter code) are in colors corresponding to their chemical properties.

Supplementary Table 1: Reagents and Tools Table

Reagent or tool	Source	Identifier
**Antibodies**		
Anti-FLAG M1 antibody	Sigma	F3040
Alexa Fluor 555 anti-mouse antibody	ThermoFisher Scientific	A31570
**Buffers**		
DMEM	ThermoFisher Scientific	10564-011
FluoroBrite DMEM	ThermoFisher Scientific	A1896701
Neurobasal Plus	ThermoFisher Scientific	A3582901
B-27™ Plus	ThermoFisher Scientific	A3582801
HBSS	ThermoFisher Scientific	14025050
Tag-lite buffer	Revvity	LABMED
**Chemicals, peptides and recombinant proteins**		
Dopamine	Sigma	H8502
Quinpirole	Sigma	Q102
Haloperidol	Sigma	H1512
Aripiprazole	Sigma	SML0935
Docosahexaenoic acid, DHA	Sigma	D2534
Docosapentaenoic acid, DPA	Sigma	18566
Behenic acid, BA	Sigma	216941
Alexa Fluor 568-Tfn	ThermoFisher Scientific	T23365
Isoproterenol	Sigma	I6504
SKF81297	Tocris	1447
DPDPE	Sigma	5.08160
U69593	Sigma	U103
DAMGO	MedChemExpress	HY-P0210
AVP	Bachem	H-1780

TetraSpeck microspheres	ThermoFisher	T7280
Fluoromount-G	ThermoFisher	00-4958-02
**Experimental models: cell lines**		
HEK-293 cells	ECACC	12022001
**Oligonucleotides**		
SEP-D2R WT FW (5’-3’)	tgttcgccgactacaagaccggtgatgacgccatggatcc	
SEP-D2R WT REV (5’-3’)	ggatccatggcgtcatcaccggtcttgtagtcggcgaaca	
D2R(S147,148A) FW (5’-3’)	tacaatacgcgctacgccgccaagcgccgggtcacc	
D2R(S147,148A) REV (5’-3’)	ggtgacccggcgcttggcggcgtagcgcgtattgta	
**Recombinant DNA**		
SEP-D2R WT	This study	
FLAG-D2R WT	Prof. Jonathan Javitch, Columbia University	
SNAP-D2R WT	Revvity	
SEP-D2R(S147,148A)	This study	
FLAG- D2R(S147,148A)	This study	
SNAP- D2R(S147,148A)	This study	
TfR-Lime		[47]
FLAG-β2AR	Prof. Aylin Hanyaloglu, Imperial College London	
SEP-β2AR		[78]
SNAP-β2AR	Revvity	
SNAP-β1AR	Revvity	
SNAP-D1R	Revvity	
SNAP-DOR	Revvity	
SNAP-KOR	Revvity	
SNAP-MOR	Revvity	
SNAP-V2R	Revvity	

βarrestin2-mCherry	Dr. Stefano Marullo, Institut Cochin	[75]
βarrestin2	Revvity	
GRK2	Dr. Philippe Marin, Institut Genomique Fonctionnelle, Montpellier	
**Software and algorithms**		
Metamorph 7.10	https://www.moleculardevices.com/ products/cellular-imaging-systems/ acquisition-and-analysis-software/ metamorph-microscopy	N/A
MATLAB 2018b	https://fr.mathworks.com	N/A
Custom MATLAB scripts	Scission_analysis [45]	MATLAB Central File Exchange 72744- scission_analysis
ImageJ 1.53c	http://www.imagej.nih.gov/ij	N/A
Galaxie	Varian	
**Kits**		
QuickChange Mutagenesis kit	Agilent	200519

Supplementary Table 2: statistical tests

Figure	Test	Post test	Comparison i	Comparison ii	Comparison iii	Comparison iv
Figure 1B	One-way ANOVA	Dunnett's multiple comparisons	DHA 10 vs DHA 30 p = 0.3300	DHA 10 vs DPA 60 p < 0.0001	DHA 10 vs DPA 120 p < 0.0001	
	One-way ANOVA	Dunnett's multiple comparisons	DPA 10 vs DPA 30 p > 0.9999	DPA 10 vs DPA 60 p = 0.9992	DPA 10 vs DPA 120 p < 0.0001	
	One-way ANOVA	Dunnett's multiple comparisons	BA 10 vs BA 30 p = 0.9723	BA 10 vs BA 60 p = 0.5912	BA 10 vs BA 120 p = 0.9993	
Figure 1C	One-way ANOVA	Dunnett's multiple comparisons	EtOH vs DHA p = 0.9893	EtOH vs DPA p = 0.5797	EtOH vs BA p = 0.9998	
Figure 1D	One-way ANOVA	Dunnett's multiple comparisons	EtOH vs DHA p = 0.0003	EtOH vs DPA p = 0.0002	EtOH vs BA p = 0.2697	
Figure 1E	One-way ANOVA	Dunnett's multiple comparisons	EtOH vs DHA p = 0.8748	EtOH vs DPA p = 0.9546	EtOH vs BA p = 0.0031	
Figure 1F	One-way ANOVA	Dunnett's multiple comparisons	EtOH vs DHA p < 0.0001			
Figure 1G	One-way ANOVA	Dunnett's multiple comparisons	EtOH vs DPA p < 0.0001			
Figure 1H	One-way ANOVA	Dunnett's multiple comparisons	EtOH vs BA p < 0.0001			
Figure 1I	One-way ANOVA	Dunnett's multiple comparisons	EtOH vs DHA p = 0.0016	EtOH vs DPA p < 0.0001	EtOH vs BA p = 0.6467	
Figure 2E	Two-way ANOVA	Sidak’s multiple comparisons	Dopa EtOH vs DHA p < 0.0001	Dopa EtOH vs DPA p < 0.0001	QPL EtOH vs DHA p < 0.0001	QPL EtOH vs DPA p < 0.0001
Figure 2F	Two-way ANOVA	Sidak’s multiple comparisons	Dopa EtOH vs DHA p = 0.9802	Dopa EtOH vs DPA p = 0.9989	QPL EtOH vs DHA p = 0.0005	QPL EtOH vs DPA p = 0.0003
Figure 2H	Two-way ANOVA	Sidak’s multiple comparisons	Dopa EtOH vs BA p = 0.2649	QPL EtOH vs BA p = 0.9856		
Figure 3B	One-way ANOVA	Dunnett's multiple comparisons	-Dopa EtOH vs DHA p = 0.0121	-Dopa EtOH vs DPA p = 0.0087	+Dopa EtOH vs DHA p < 0.0001	+Dopa EtOH vs DHA p < 0.0001
Figure 3D	One-way ANOVA	Dunnett's multiple comparisons	-QPL EtOH vs DHA p = 0.1444	-QPL EtOH vs DPA p = 0.0720	+QPL EtOH vs DHA p < 0.0001	+QPL EtOH vs DPA p < 0.0001
Figure 3F	One-way ANOVA	Dunnett's multiple comparisons	-QPL EtOH vs DHA p > 0.9999	-QPL EtOH vs DPA p = 0.0633	+QPL EtOH vs DHA p < 0.0001	+QPL EtOH vs DPA p < 0.0001
Figure 4D	One-way ANOVA	Dunnett's multiple comparisons	-Iso EtOH vs DHA p = 0.4814	-Iso EtOH vs DPA p = 0.7860	+Iso EtOH vs DHA p = 0.1516	+Iso EtOH vs DPA p = 0.7110
Figure 5A	One-way	Dunnett's	EtOH vs DHA	EtOH vs DPA		

	ANOVA	multiple comparisons	p = 0.0005	p = 0.9187		
Figure 5B	One-way ANOVA	Dunnett's multiple comparisons	EtOH vs DHA<br>p = 0.7261	EtOH vs DPA<br>p < 0.0001		
Figure 5C	One-way ANOVA	Dunnett's multiple comparisons	EtOH vs DHA<br>p = 0.8920	EtOH vs DPA<br>p = 0.0296		
Figure 5D	One-way ANOVA	Dunnett's multiple comparisons	EtOH vs DHA<br>p = 0.0659	EtOH vs DPA<br>p = 0.9993		
Figure 5F	One-way ANOVA	Dunnett's multiple comparisons	-QPL<br>EtOH vs DHA<br>p = 0.1055	-QPL<br>EtOH vs DPA<br>p = 0.8750	+QPL<br>EtOH vs DHA<br>p = 0.0169	+QPL<br>EtOH vs DPA<br>p = 0.0175
Figure 6C	One-way ANOVA	Dunnett's multiple comparisons	EtOH vs DHA<br>p = 0.7943	EtOH vs DPA<br>p = 0.9786		
Figure 6G	One-way ANOVA	Dunnett's multiple comparisons	EtOH vs DHA<br>p = 0.0160	EtOH vs DPA<br>p = 0.0441		
Figure 7E	One-way ANOVA	Dunnett's multiple comparisons	EtOH vs DHA<br>p = 0.1400	EtOH vs DPA<br>p = 0.9095		
Figure 7F	One-way ANOVA	Dunnett's multiple comparisons	EtOH vs DHA<br>p = 0.0327	EtOH vs DPA<br>p = 0.0352		
Figure 7G	One-way ANOVA	Dunnett's multiple comparisons	EtOH vs DHA<br>p = 0.9646	EtOH vs DPA<br>p = 0.7626		
Figure 7H	One-way ANOVA	Dunnett's multiple comparisons	EtOH vs DHA<br>p = 0.8391	EtOH vs DPA<br>p = 0.9827		
Figure 7I	One-way ANOVA	Dunnett's multiple comparisons	EtOH vs DHA<br>p = 0.0285	EtOH vs DPA<br>p = 0.0068		
Figure 8B	Unpaired t test	NA	-QPL WT vs S147,148A<br>p = 0.9209	+QPL WT vs S147,148A<br>p = 0.3732		
Figure 8C	One-way ANOVA	Dunnett's multiple comparisons	-QPL<br>EtOH vs DHA<br>p = 0.7938	-QPL<br>EtOH vs DPA<br>p = 0.9551	+QPL<br>EtOH vs DHA<br>p = 0.3530	+QPL<br>EtOH vs DPA<br>P = 0.0804
Figure 8D						
Figure 8E	One-way ANOVA	Dunnett's multiple comparisons	EtOH vs DHA<br>p = 0.8962	EtOH vs DPA<br>p = 0.9543		
Figure 8F	One-way ANOVA	Dunnett's multiple comparisons	EtOH vs DHA<br>p = 0.7381	EtOH vs DPA<br>p = 0.2948		
Figure 8G	One-way ANOVA	Dunnett's multiple comparisons	EtOH vs DHA<br>p = 0.8634	EtOH vs DPA<br>p = 0.9775		

**Supp Figure**	**Test**	**Post test**	**Comparison i**	**Comparison ii**	**Comparison iii**	
Figure	Two-way	Dunnett's	40’	40’ +QPL	40’ +QPL	40’ +QPL

S1B	ANOVA	multiple comparisons	D2R no QPL vs +QPL<br>p = 0.4539	D2R vs D2R/bArr<br>p = 0.0100	D2R vs D2R/GRK2<br>p = 0.0005	D2R vs D2R/GRK2/bArr<br>p < 0.0001
Figure S2G	Two-way ANOVA	Dunnett's multiple comparisons	45'<br>EtOH vs DHA<br>p = 0.4467<br>EtOH vs DPA<br>p = 0.1737	60'<br>EtOH vs DHA<br>p = 0.5838<br>EtOH vs DPA<br>p = 0.2762	75'<br>EtOH vs DHA<br>p = 0.6964<br>EtOH vs DPA<br>p = 0.4085	
Figure S2H	Two-way ANOVA	Dunnett's multiple comparisons	45'<br>EtOH vs DHA<br>p = 0.0119<br>EtOH vs DPA<br>p = 0.0052	60'<br>EtOH vs DHA<br>p = 0.0131<br>EtOH vs DPA<br>p = 0.1686	75'<br>EtOH vs DHA<br>p = 0.0451<br>EtOH vs DPA<br>p = 0.1074	
Figure S2I	Two-way ANOVA	Dunnett's multiple comparisons	45'<br>EtOH vs DHA<br>p = 0.0065<br>EtOH vs DPA<br>p = 0.0060	60'<br>EtOH vs DHA<br>p = 0.0068<br>EtOH vs DPA<br>p = 0.0034	75'<br>EtOH vs DHA<br>p = 0.0077<br>EtOH vs DPA<br>p = 0.0077	
Figure S2J	Two-way ANOVA	Dunnett's multiple comparisons	45'<br>EtOH vs DHA<br>p = 0.0003<br>EtOH vs DPA<br>p < 0.0001	60'<br>EtOH vs DHA<br>p < 0.0001<br>EtOH vs DPA<br>p = 0.0009	75'<br>EtOH vs DHA<br>p = 0.0015<br>EtOH vs DPA<br>p = 0.0008	
Figure S3C	One-way ANOVA	Dunnett's multiple comparisons	-QPL<br>EtOH vs DHA<br>p=0.8143<br>EtOh vs DPA<br>p=0.6439	+QPL<br>EtOH vs DHA<br>p=0.9266<br>EtOh vs DPA<br>p=0.3735		
Figure S3D	One-way ANOVA	Dunnett's multiple comparisons	+QPL<br>EtOH vs DHA<br>p=0.2609<br>EtOh vs DPA<br>p=0.1933			
Figure S4C	One-way ANOVA	Dunnett's multiple comparisons	EtOH vs DHA<br>p = 0.0718	EtOH vs DPA<br>p = 0.1950		
Figure S4G	One-way ANOVA	Dunnett's multiple comparisons	EtOH vs DHA<br>p = 0.0002	EtOH vs DPA<br>p < 0.0001		
Figure S5B	One-way ANOVA	Dunnett's multiple comparisons	-QPL<br>EtOH vs DHA<br>p=0.1661<br>EtOH vs DPA<br>p=0.9452	+QPL 10'<br>EtOH vs DHA<br>p=0.0002<br>EtOH vs DPA<br>p=0.0004	+QPL 90'<br>EtOH vs DHA<br>p=0.0339<br>EtOH vs DPA<br>p=0.0023	
Figure S5C	Kruskal-Wallis test	Dunn's multiple comparisons test	EtOH vs DHA<br>p=0.9306<br>EtOH vs DPA<br>p>0.9999	EtOH vs DHA<br>p=0.0366<br>EtOH vs DPA<br>p=0.0713		
Figure	One-way	Dunnett's	EtOH vs DHA	EtOH vs DPA		

S6B	ANOVA	multiple comparisons	p = 0.9988	p = 0.2554		
Figure S6C	One-way ANOVA	Dunnett's multiple comparisons	EtOH vs DHA p = 0.5464	EtOH vs DPA p = 0.6296		
Figure S6D	One-way ANOVA	Dunnett's multiple comparisons	EtOH vs DHA p = 0.7851	EtOH vs DPA p = 0.4346		
Figure S7B	One-way ANOVA	Dunnett's multiple comparisons	EtOH vs DHA p = 0.8336	EtOH vs DPA p = 0.9344		
Figure S7C	One-way ANOVA	Dunnett's multiple comparisons	EtOH vs DHA p = 0.4718	EtOH vs DPA p = 0.4919		
Figure S7D	One-way ANOVA	Dunnett's multiple comparisons	EtOH vs DHA p = 0.4129	EtOH vs DPA p = 0.4279		
Figure S7E	One-way ANOVA	Dunnett's multiple comparisons	EtOH vs DHA p = 0.9384	EtOH vs DPA p = 0.6238		
Figure S8A	One-way ANOVA	Dunnett's multiple comparisons	WT DHA vs WT DPA p = 0.0107	Mut DHA vs mut DPA p = 0.0018		
