# Supplementary material for: Membrane lipid poly-unsaturation selectively affects dopamine D2 receptor endocytosis
Source: Nat Commun. 2026 May 20;17:6661. doi: 10.1038/s41467-026-73057-5 (PMC13381900; doi:10.1038/s41467-026-73057-5)
Supplement: Supplementary file 2 — Description of Additional Supplementary Files [file 41467_2026_73057_MOESM2_ESM.pdf]

Description of Additional Supplementary Files

File name: Supplementary Data 1
Alignment of residues in regions TM3, ICL2 and TM4 of the 200 human non-orphan, non-opsin class A GPCRs. Alignment performed with GPCRdb (gpcrdb.org) Left, agonists and names of human GPCRs (UniProt). Residues (in one letter code) are in colors corresponding to their chemical properties.

File name: Supplementary Movie 1
Description: Recruitment and clustering of SEP-D2R (left) and β-Arrestin2-mCherry (right) before and during application of QPL observed with TIRF microscopy in a cell treated with ethanol carrier.

File name: Supplementary Movie 2
Description: Recruitment and clustering of SEP-D2R (left) and β-Arrestin2-mCherry (right) before and during application of QPL observed with TIRF microscopy in a cell treated with DHA.

File name: Supplementary Movie 3
Description: Recruitment and clustering of SEP-D2R (left) and β-Arrestin2-mCherry (right) before and during application of QPL observed with TIRF microscopy in a cell treated with DPA.

File name: Supplementary Movie 4
Description: Formation of endocytic vesicles detected with the ppH assay before, during and after application of QPL. SEP-D2R at extracellular pH 7.4 (left) and pH 5.5 (middle), β-Arrestin2-mCherry at pH 5.5 (right).

File name: Supplementary Movie 5
Description: Formation of endocytic vesicles detected with the ppH assay. TfR-Lime at extracellular pH 7.4 (left) and pH 5.5 (right).

File name: Supplementary Movie 6
Description: Formation of endocytic vesicles detected with the ppH assay, before, during and after application of Isoproterenol (ISO). SEP-B2AR at extracellular pH 7.4 (left) and pH 5.5 (middle), β-Arrestin2-mCherry at pH 5.5 (right).
