# Supplementary material for: Membrane lipid poly-unsaturation selectively affects dopamine D2 receptor endocytosis
Source: Nat Commun. 2026 May 20;17:6661. doi: 10.1038/s41467-026-73057-5 (PMC13381900; doi:10.1038/s41467-026-73057-5)
Supplement: Supplementary file 11 — Transparent Peer Review file [file 41467_2026_73057_MOESM11_ESM.pdf]

nature portfolio

Peer Review File

Membrane lipid poly-unsaturation selectively affects dopamine D2 receptor endocytosis

Corresponding Author: Dr David Perrais

This file contains all reviewer reports in order by version, followed by all author rebuttals in order by version.

Version 0:

Reviewer comments:

Reviewer #1

(Remarks to the Author)
The study by Sposini and colleagues centers on the question if alterations in the plasma membrane lipid composition affect agonist-driven regulation of the dopamine D2 receptor (D2R). Decreases in polyunsaturated fatty acids (PUFA) have been observed in psychiatric diseases, which are also frequently linked to altered D2R activity. The presented work builds on a previous study by Jobin et al (ref 25), where the authors discovered that an enrichment in the polyunsaturated fatty acids DHA and DPA can alter D2R ligand binding affinities, G protein signaling, and beta-arrestin recruitment. Sposini and colleagues now find that increased levels of DHA and DPA (PUFAs with 22 carbons but different positions of the double bond) decrease D2R internalization and lower beta-arrestin recruitment in HEK293 cells. Furthermore, they identify two serine residues in D2R's ICL2 that are involved in mediating the modulatory effects of PUFAs. The authors also show that enrichment of DHA and DPA does not alter the internalization of seven other GPCRs or the transferrin receptor. While studying the impact of lipids on GPCR signal transduction is highly relevant, especially in the context of neuromodulatoy GPCRs, the insights provided by the presented work remain incremental. Many open questions remain, which are linked to the impact of PUFA-mediated regulation on D2R signaling and trafficking, the experimental conditions used, and the discrepancy between the presented results and earlier work published by some of the co-authors.

Specific comments:
Figure 1 describes the experimental setup and the successful incorporation of the exogenous PUFAs (DHA and DPA) or the control saturated fatty acid BA into HEK293 cells. Throughout the manuscript, the authors use the same enrichment protocol (30 µM exogenous lipid, 24 h incubation before analysis). However, the system would likely allow to carefully titer the amount of the respective fatty acids by varying the exogenously added lipid concentration or duration of incubation. Such a titration would enable the authors to establish at what molar percentage DHA and DPA significantly change D2R internalization (subsequent figures). This would allow meaningful insight with regards to physiological studies that have investigated PUFA levels in the (diseased) brain.

Figures 2 and 3 show that DHA and DPA enrichment decreases dopamine- and quinpirole-mediated D2R internalization in HEK293 cells (both figures could therefore be combined into a single figure). While the presented results are convincing, it is not clear why the authors co-express GRK2 and beta-arrestin2 in all their experiments. What is the rationale for this, and would the effect hold true at endogenous levels of these regulators / transducers? This is important since overexpression of GRK2 and beta-arrestin2 are well known to alter the extent of GPCR phosphorylation and endocytosis. In addition, it is important to test if the DHA and DPA driven decrease in D2R internalization is observed in other cell lines or cell types (ideally neuronal), specifically following the author's introduction and discussion on D2R's role in neurotransmission.

Figure 4 shows that DHA and DPA enrichment do not alter the constitutive endocytosis of the transferrin receptor and does not affect the ligand-driven internalization of seven other GPCRs. Here again, GRK2 and beta-arrestin2 are over-expressed, which represents an artificial experimental system. Furthermore, it is not clear how these receptors were chosen. Why were D3R, nociceptin receptor (NOP), lysophospholipid receptor S1PR5 and melatonin receptor MTR1a not tested, which appear to have consecutive serines in the ICL2 region similar to D2R (Figure 5 and manuscript discussion). These should be included in the analyses to make broader conclusions about the ICL2 involvement in PUFA-mediated regulation.

Figure 5&6 shows that beta-arrestin recruitment to D2R clusters is weaker upon DHA and DPA enrichment. This finding is consistent with the decrease in D2R internalization (Fig. 2&3) and the known role for beta-arrestin in D2R internalization.

However, in a previous study, the authors reported the exact opposite effect: In the Jobin et al. paper (ref 25), an identical approach was taken to enrich membranes in DHA and DPA and here, the arrestin recruitment to D2R was significantly increased (ref 25, Fig. 3). The authors need to mention, discuss, and experimentally address the discrepancy in findings. Furthermore, a lot of additional mechanistic questions arise, which would need to be experimentally addressed to delineate what the mechanistic impact of altered PUFA levels is: What is the effect of DHA and DPA enrichment on G protein coupling to D2R? Is the GRK2 recruitment to and phosphorylation of D2R altered upon DHA and DPA enrichment? Are the receptor clusters co-localized with clathrin coated pits (CCPs)? And if so, why are receptor cluster unchanged if the link to the clathrin coated machinery, i.e. beta-arrestin, is reduced? How do the changes in ligand affinity noted in the earlier work (ref 25, Fig. 1) relate to the findings of this study?

Figure 7 shows that internalization of the ICL2 serine double mutant of D2R is not affected by DHA and DPA enrichment, distinct to the wild-type receptor. While this is potentially interesting, the functionality of the mutated receptor needs to be tested. Do the mutations affect ligand affinity and G protein coupling? And see above comment on Fig. 4: is the serine motif, detected by the authors in other GPCRs, predictive for regulation by DHA and DPA?

In sum, the finding of Sposini and colleagues that PUFAs impact D2R internalization and beta-arrestin recruitment are interesting. Yet several additional experiments outlined above are needed to make this a comprehensive study with new mechanistic insights.

Reviewer #2

(Remarks to the Author)
This interesting study uses live and fixed cell trafficking assays to demonstrate that incorporation of poly-unsaturated (PUFAs), but not saturated, free fatty acids into membrane phospholipids reduce agonist induced internalization of the D2 dopamine receptor. Pulsed pH live microscopy was used to demonstrate that the reduced internalization by incorporation of free fatty acid was accompanied by a blunted recruitment of Beta arrestin for the D2R. The manuscript sets out well-designed experiments and the observations are generally supported by their data. A particular strength is the validation of incorporation of FFA's in the plasma membrane. This builds on previous work looking at the modulation of D2R signalling and D2R-mediated behaviors by PUFAs. There does seem to be a discrepancy between the effect of PUFAs on arrestin recruitment between this study and the previous study (enhanced in the previous study, inhibited in this one) (Jobin et al. PMID: 37550407).

The role of PUFAs in modulating D2R signalling has been studied before by the authors, with evidence to suggest that PUFAs modulate dopamine-controlled behaviors (Ducrocq et al. PMID: 32142670. and Jobin et al. PMID: 37550407). The present study does not extend to assess the physiological, pathophysiological or therapeutic role of the observations (except from referring the relation between FFAs and disease). The link between the findings of this study (reduced internalization and arrestin recruitment) and their previous work are not clear, particularly given the above discrepancy. The mechanism by which PUFAs might inhibit arrestin recruitment and subsequently internalization remains undefined although the authors provide some ideas in their discussion.

The authors show that the effect of the PUFAs to restricted to the D2R as compared to a battery of other selected GPCRs. They provide a rationale for this selective action and suggest a number of other receptors that might be sensitive to PUFAs. Confirming this hypothesis would broaden the interest and impact of the paper along with providing more mechanistic insight.

Specific comments are as follows:

There appears to be a discrepancy between the authors’ findings in this paper, namely that PUFAs inhibit D2R internalization and the recruitment of arrestin, and their previous study which illustrates that enrichment of PUFAs robustly increase agonist stimulated arrestin recruitment (Jobin et al. PMID: 37550407). The authors should explain this discrepancy.

The authors suggest that D2R internalization is dependent on GRK2 and B arrestin2. While there is certainly evidence of B arrestin 2 and GRK2 overexpression potentiating D2R internalization, the essential role of arrestin was not conclusively demonstrated with knock out/knock down of arrestin (https://doi.org/10.1074/jbc.M106728200). The recent work by Patel and colleagues (PMID: 39638791) demonstrates that, both in a HEK293 cell background and in striatal neurons, D2R internalization is independent of B arrestin but instead driven by the cytoskeletal motor myosin VI in a process that also involves a PDZ domain within the C-terminal tail of D2R, GIPC and is regulated by the intracellular loop 3 of the D2R. From that study it seems like that the level of D2R internalisation is unaffected by the knockout of B arrestin. Can the authors reconcile their findings with this study? Might PUFAs instead regulate this process? The authors could provide further insight regarding these two different mechanisms. The repetition of their findings in the CRISPR B arrestin K/O HEK cell lines would be a useful experiment (PMID: 29362459).

In the light of the discrepancy with their previous work regarding the effect of PUFAs on B arrestin recruitment, and the different mechanisms proposed to underlie agonist stimulated internalization of the D2R, the mechanistic gains from this study are unclear. It remains unclear exactly how PUFAs might modulate D2R internalisation and what how the effect on B arrestin recruitment to the D2R links to receptor internalization. It is also not clear from their data what effect PUFAs have on

D2R receptor function. From their previous study, Jobin et al. PMID: 37550407, enrichment of PUFAs has no effect on G protein signalling as measured in a cAMP and G protein activation assay. If arrestin recruitment and/or receptor internalization is inhibited/enhanced then we might expect some change in receptor G protein signalling?

The use of the mutant receptor to ablate PUFA sensitivity of the receptor is nice but what exactly these residues do is not clear. The authors propose that enriching PUFAs will cause ICL2 to be buried in the membrane but it is not clear what effect this will then have. Which cytosolic proteins will no longer interact with the D2R and why? Are these serines phoshphorylated. Do you see reduced phosphorylation of these residues in the presence of PUFAs? Do PUFAs impact the recruitment of GRKs? Can the authors measure how PUFA changes its interaction with GIPC? It is a nice observation that relatively few GPCRs have the double serines in ICL2 and it would certainly add to the study to demonstrate that some of the others that have this motif are also sensitive to PUFAs. If you add this motif to a non-PUFA sensitive GPCR, do you confer sensitivity? This would provide more evidence of the proposed mechanism.

There is a question of how the insights gained in this study in a HEK293 cell background will translate into a disease-relevant cell background. Is there not the possibility that such observations will be cell dependent? Could the authors validate their findings in, for example, striatal neuronal cultures to demonstrate the same impact of PUFAs on D2R internalization. Why did the authors choose to overexpress both GRK2 and B2 arrestin in this study and what impact this has on their observations? Did they try analogous experiments without this over expression?

D2R internalization is measured over an 80-minute timescale, and significant internalisation appears to take about 30 minutes. How does this relate to the relatively transient release and uptake of dopamine at a synapse?

In their analysis of microscopy it appears that the authors combine the data from 3 independent experiments (of x number of cells per condition) together before analysing with ANOVA. With all data points included together it would seem there is the possibility that data from one experimental repeat might unduly influence this analysis. Why weren't mean effects of each experimental repeat taken and then these combined (so three mean values) and compared across the different conditions?

Reviewer #3

(Remarks to the Author)
Over the past year, the authors have substantially revised the manuscript and thoroughly addressed my previous concerns regarding the interpretation of their findings. In the earlier version, the narrative could be interpreted as suggesting that blunted D2R internalization reflected cellular dysfunction, implying that increased PUFA levels negatively impact cellular physiology. In the current version, however, the authors have clarified that the observed reduction in D2R internalization is not inherently beneficial or detrimental, but rather a direct consequence of altered membrane PUFA content. This neutral, mechanistic framing is well-supported by the data. Only a few minor points remain that should be addressed or clarified before the manuscript is suitable for publication:

1. I appreciate the inclusion of Figure 1 in the main text and recognize the effort to clarify the effects of DHA, DPA, and BA on fatty acid composition. It is clear that DHA and DPA increase the proportion of polyunsaturated fatty acids (PUFAs) while reducing saturated fatty acids (SFAs), whereas BA increases SFAs with minimal impact on PUFA levels. However, the data remain somewhat puzzling in the context of mass conservation. If total fatty acids (Total FA) are composed of PUFAs and SFAs, an inverse relationship between these two categories would be expected. Yet this relationship is not apparent in Figure 1C. Additionally, the combined percentages from Figures 1B and 1C do not appear to sum to 100%. For instance, DHA treatment shows approximately 15% PUFA in Figure 1B and 35% PUFA in Figure 1C - totaling only 50%, which is unexpectedly low. Does this imply that monounsaturated fatty acids (MUFAs) constitute a substantial portion of the Total FA and are not explicitly represented in these panels? Clarifying this apparent discrepancy and the role of MUFAs in the overall fatty acid balance is necessary for accurate interpretation of the data.

2. Figures 1D-F clearly show that incubation with DHA, DPA, and BA results in an increased abundance of the respective fatty acids in the lipidomic analysis. However, it remains unclear whether these fatty acids are truly metabolized and incorporated into cellular membranes - particularly the plasma membrane - or if they are simply retained within the cell as free fatty acids, stored in lipid droplets, or peripherally associated with membranes in a way that allows them to be extracted during sample preparation. Additional clarification is needed in the manuscript to distinguish between these possibilities and to support the conclusion that the observed lipidomic changes reflect bona fide membrane incorporation.

Version 1:

Reviewer comments:

Reviewer #1

(Remarks to the Author)
The revised manuscript by Sposini and colleagues contains several new datasets that strengthen the conclusion that PUFA levels affect agonist-driven D2R internalization (in GRK and beta-arrestin overexpression conditions, see below). Also, the rationale for using the specific PUFA enrichment protocol is now clearer (revised Fig. 1), and the new results showing reduced D2R internalization upon increasing PUFA levels in cultured neurons represent a valuable addition (Fig. 5).

Based on my previous comments and the newly added data, the following points remain:
1. The authors now show that β-arrestin and GRK2 overexpression strongly enhances agonist-driven D2R internalization, which they use as the rationale for employing these conditions throughout the study (Fig. S1). However, given that agonist-driven D2R internalization is still detectable in cells not overexpressing these regulators (2-fold over basal), and considering previous work establishing that D2R internalization occurs independently of β-arrestin (Patel et al., 2024), it is important to test whether PUFA levels affect D2R internalization in cells not overexpressing GRK2 and β-arrestin. This straight-forward experiment is both meaningful and important to for the broader validity of the findings.
2. The hypothesis that the two-serine motif in the ICL2 of GPCRs contributes to PUFA-regulated internalization was not tested for other GPCRs. Plasmids for GPCR constructs are readily available (e.g., via Addgene), making it surprising that this was not investigated, particularly given that two reviewers raised this point.
3. The new data on GIPC recruitment do not represent a reliable or conclusive addition to the manuscript (Fig. S6), and I recommend removing this section for the following reasons: First, GIPC recruitment was assessed in cells overexpressing GRK and β-arrestin, which are likely to sterically compete with GIPC binding, especially given their overexpression. Second, no convincing agonist-driven GIPC recruitment above background was detected (Fig. S6E), making it impossible to conclude that GIPC recruitment is unaffected by changes in PUFA levels, as stated on p. 13.
4. I appreciate that the authors attempted to address discrepancies between the present study and their previous work by examining β-arrestin recruitment to D2R clusters at longer time points following PUFA manipulation. However, β-arrestin colocalization with D2R clusters at 90 minutes is not evident based on the images provided (neither EtOH control, nor DHA and DPA conditions) (Fig. S5). It would be important to show the individual β-arrestin and D2R images in grayscale, in addition to the merged images, to allow a clearer assessment of the validity of these data.
5. In the discussion, I find this following paragraph problematic and strongly recommend rewording or deletion:
“However, activation of β-arrestin by GPCRs can occur through a recently described catalytic pathway which does not require the formation of a stable complex (ref 43,62). In this context, transient activation of membrane-associated β-arrestin by interaction with GPCRs drives its association with CCPs (ref 62). It is thus possible to decouple D2R recruitment from β-arrestin recruitment to CCPs, resulting in DHA or DPA membrane enrichment only affecting D2R endocytosis and not its clustering at CCPs.” The cited studies show that clustering of β-arrestin in CCPs can occur independently of GPCR recruitment to CCPs, however, they do not demonstrate that GPCR clustering in CCPs is decoupled from β-arrestin. This distinction is important, as the authors use these citations to support the possibility that D2R clustering in CCPs is decoupled from β-arrestin, which is not justified by the referenced work.

Reviewer #2

(Remarks to the Author)
In their revised manuscript, Sponsini and colleagues have added a significant amount of additional data and discussion to address almost all of my comments. I have only minor comments remaining.

They have provided additional data and explanation around the discrepancy between the findings of this paper and that of Jobin et al. in terms of the effect of PUFAs on arrestin recruitment to the D2R. I think it would be useful for the reader to have a bit more detail explaining explicitly the nature of the previous experiment (whole cell assay of arrestin recruitment that does not distinguish between the D2R at the PM and at intracellular compartments) in addition to the difference in agonist incubation time. I think the authors are implying that the previous experiment in Jobin et al. is is likely thaat the measurment is of arrestin recruitment to D2Rs within the endosome. If so I think this is worth stating explicitly.

From a mechanistic point of view can the authors speculate why after 90 minutes they do see an increase in arrestin recruitment to the D2R in intracellular compartments with the addition of PUFAs, what changes occur to reverse the initial effect of decreasing arrestin recruitment which is presumably occurring predominantly at the PM?

The addition of data for the internalisation of Flag-D2R in cortical primary neurons is a nice addition and shows that the effects in these cells match those seen in their HEK experiments. The additional data around clustering at CCPs and the experiments looking at GIPC recruitment are also useful additions.

There are some additional questions that the authors were not able to address but I agree that such questions, such as the impact of PUFAs on D2R signalling lie beyond the scope of this manuscript.

Reviewer #3

(Remarks to the Author)
The authors have thoroughly addressed my first comment regarding mass balance on the lipid tails directly in the revised manuscript.

With respect to my second concern about interpreting the lipidomic shifts after DHA/DPA/BA incubation, the authors provided a thorough and well-reasoned explanation in their response, including relevant literature. However, this clarification is not reflected in the manuscript. As written, the manuscript infers membrane enrichment from whole-cell phospholipid measurements without explicitly addressing whether these changes reflect plasma membrane incorporation versus other cellular lipid pools. This clarification is important for the strength of the mechanistic interpretation and should be incorporated into the manuscript.

I therefore ask that the authors incorporate a brief version of their explanation into the manuscript (Results and/or

Discussion), with appropriate citations and an explicit statement of the assumptions regarding plasma membrane enrichment.

Version 2:

Reviewer comments:

Reviewer #1

(Remarks to the Author)
I thank the authors for addressing the remaining points with additional data and text modifications. I now support the publication of the manuscript.

Open Access This Peer Review File is licensed under a Creative Commons Attribution 4.0 International License, which permits use, sharing, adaptation, distribution and reproduction in any medium or format, as long as you give appropriate credit to the original author(s) and the source, provide a link to the Creative Commons license, and indicate if changes were made.
In cases where reviewers are anonymous, credit should be given to 'Anonymous Referee' and the source.
The images or other third party material in this Peer Review File are included in the article’s Creative Commons license, unless indicated otherwise in a credit line to the material. If material is not included in the article’s Creative Commons license and your intended use is not permitted by statutory regulation or exceeds the permitted use, you will need to obtain permission directly from the copyright holder.
To view a copy of this license, visit https://creativecommons.org/licenses/by/4.0/

REVIEWER COMMENTS

We have reproduced the reviewers' queries in black and written our answers in blue. We have attached the complete list of references used in these answers at the end of this document.

Reviewer #1 (Remarks to the Author):

The study by Sposini and colleagues centers on the question if alterations in the plasma membrane lipid composition affect agonist-driven regulation of the dopamine D2 receptor (D2R). Decreases in polyunsaturated fatty acids (PUFA) have been observed in psychiatric diseases, which are also frequently linked to altered D2R activity. The presented work builds on a previous study by Jobin et al (ref 25), where the authors discovered that an enrichment in the polyunsaturated fatty acids DHA and DPA can alter D2R ligand binding affinities, G protein signaling, and beta-arrestin recruitment. Sposini and colleagues now find that increased levels of DHA and DPA (PUFAs with 22 carbons but different positions of the double bond) decrease D2R internalization and lower beta-arrestin recruitment in HEK293 cells. Furthermore, they identify two serine residues in D2R's ICL2 that are involved in mediating the modulatory effects of PUFAs. The authors also show that enrichment of DHA and DPA does not alter the internalization of seven other GPCRs or the transferrin receptor. While studying the impact of lipids on GPCR signal transduction is highly relevant, especially in the context of neuromodulatoy GPCRs, the insights provided by the presented work remain incremental. Many open questions remain, which are linked to the impact of PUFA-mediated regulation on D2R signaling and trafficking, the experimental conditions used, and the discrepancy between the presented results and earlier work published by some of the co-authors.

We thank the reviewer for his/her general and positive comments. Nevertheless, we respectfully disagree with the conclusion that "…the insights provided by the presented work remain incremental." We show that the enrichment in DHA or DPA specifically affect the endocytosis of D2R without affecting the endocytosis of many other GPCRs and of the transferrin receptor. Although there were previous reports of PUFAs affecting GPCR conformations or signaling, this is the first report of alterations in trafficking of a specific GPCR, D2R. Moreover, we have addressed the apparent "discrepancy between the presented results and earlier work published by some of the co-authors" with additional experiments. Overall, we have thrived to address all the points raised and some of the many open questions.

Specific comments:

Figure 1 describes the experimental setup and the successful incorporation of the exogenous PUFAs (DHA and DPA) or the control saturated fatty acid BA into HEK293 cells. Throughout the manuscript, the authors use the same enrichment protocol (30 µM exogenous lipid, 24 h incubation before analysis). However, the system would likely allow to carefully titer the amount of the respective fatty acids by varying the exogenously added lipid concentration or duration of incubation. Such a titration would enable the authors to establish at what molar percentage DHA and DPA significantly change D2R internalization (subsequent figures). This would allow meaningful insight with regards to physiological studies that have investigated PUFA levels in the (diseased) brain.

This is a good point which needs clarification. We have used 30 µM of DHA or DPA because it was the highest concentration tested without any toxic effect. We now show in Figure 1B that incubation with 60 µM of DHA (but not DPA or BA) for 24 h already affects cell viability. With the treatment of 30 µM we get a significant enrichment of PUFAs in membranes, as shown in Figure 1.

Moreover, we now provide lipidomics data on neuronal cultures demonstrating the possibility to enrich these cells specifically with DHA or DPA, similarly to HEK cells, resulting in comparable enrichment levels (Figure 5). Of note, the PUFA levels in neuronal cultures (~20 %) are higher than in HEK-293 cells (8-14 %) but still lower than in vivo (~30%)(Bazinet and Layé, 2014). We discuss this difference in the Discussion.

Figures 2 and 3 show that DHA and DPA enrichment decreases dopamine- and quinpirole-mediated D2R internalization in HEK293 cells (both figures could therefore be combined into a single figure). While the presented results are convincing, it is not clear why the authors co-express GRK2 and beta-arrestin2 in all their experiments. What is the rationale for this, and would the effect hold true at endogenous levels of these regulators / transducers? This is important since overexpression of GRK2 and beta-arrestin2 are well known to alter the extent of GPCR phosphorylation and endocytosis. In addition, it is important to test if the DHA and DPA driven decrease in D2R internalization is observed in other cell lines or cell types (ideally **neuronal**), specifically following the author's introduction and discussion on D2R's role in neurotransmission.

We thank the reviewer for bringing up this point. We now show in Figure S1B that in HEK-293 cells transfected with SNAP-D2R alone, addition of the D2R agonist quinpirole (QPL, 10 µM) increases its internalization less than 2-fold (compared to cells without agonist). Co-transfection of cells with GRK2 (which phosphorylates D2R) and/or βarrestin2 (which binds phosphorylated D2R), increases receptor internalization 4 to 6 fold. This gives a much clearer assay to determine the effect of enrichment with fatty acids. Therefore, we used throughout this study HEK-293 co-transfected with tagged D2R, GRK2 and βarrestin2 at 1/1/1 ratio. To address the second point, we have performed internalization experiments of FLAG-D2R in rat primary cortical neurons. In these cells, addition of QPL induces the robust internalization of D2R. Importantly, we show in Figure 5E,F that treatment with DHA or DPA decreases D2R internalization, consistent with the results obtained with HEK-293 cells.

Figure 4 shows that DHA and DPA enrichment do not alter the constitutive endocytosis of the transferrin receptor and does not affect the ligand-driven internalization of seven other GPCRs. Here again, GRK2 and beta-arrestin2 are over-expressed, which represents an artificial experimental system. Furthermore, it is not clear how these receptors were chosen. Why were D3R, nociceptin receptor (NOP), lysophospholipid receptor S1PR5 and melatonin receptor MTR1a not tested, which appear to have consecutive serines in the ICL2 region similar to D2R (Figure 5 and manuscript discussion). These should be included in the analyses to make broader conclusions about the ICL2 involvement in PUFA-mediated regulation.

We hope we answered the reviewer's first point in the answer above.

For the choice of the other 7 GPCRs, we decided to look at various members of class A GPCRs which are similar to or differ from D2R in the following properties:

- δ, µ and κ opioid receptors as they are, as D2R, also coupled to Gi;
- D1R, as it is another member of Dopamine receptor subfamily;
- Gs-coupled receptors that are considered prototypical GPCRs, such as β1-adrenergic receptor, β2 adrenergic receptor and vasopressin receptor 2. Moreover, these three receptors where chosen as they internalize through different mechanisms: β1-adrenergic receptor through clathrin-independent endocytosis, β2 adrenergic receptor through CME,

and V2R displays a robust and sustained co-localization with β-arrestin not only at the plasma membrane but also at endosomes. Finally, V2R was also chosen as it also couples to Gq/11.

Regarding the receptors that we have not tested, we have tried to assess the internalization of D3R with the DERET assay. However, as shown before (Kim et al., 2001), this receptor does not internalize, even with co-expression of GRK2 and βarrestin2. Therefore, we could not test the effect of PUFA enrichment. Regarding the other receptors, we either could not get good expression levels in HEK-293 cells (NOP) or we did not have the constructs at hand (S1PR5, MTR1a). Nevertheless, we clearly show that the ligand-induced internalization of D2R is specifically sensitive to the level of DHA or DPA in membranes and we do not make broader conclusions on the role of ICL2, but only propose it as a putative sequence for the regulation of other GPCRs by PUFAs.

Figure 5&6 shows that beta-arrestin recruitment to D2R clusters is weaker upon DHA and DPA enrichment. This finding is consistent with the decrease in D2R internalization (Fig. 2&3) and the known role for beta-arrestin in D2R internalization. However, in a previous study, the authors reported the exact opposite effect: In the Jobin et al. paper (ref 25), an identical approach was taken to enrich membranes in DHA and DPA and here, the arrestin recruitment to D2R was significantly increased (ref 25, Fig. 3). The authors need to mention, discuss, and experimentally address the discrepancy in findings.

We thank the reviewer for bringing up this important point. Indeed, the data obtained here seem at odds with the data reported previously in (Jobin et al., 2023). However, there are two key differences between the two studies. First, Jobin and co-workers assessed β-arrestin recruitment to D2R was with PathHunter assay in CHO cells. This assay measures the accumulation of the signal given by D2R-β-arrestin interaction, regardless of the location within the cell and the dynamics of this interaction. In the present study, which focusses on the endocytosis of D2R, we use light microscopy approaches to assess, in HEK293 cells, the recruitment of β-arrestin to D2R clusters and endocytic vesicles in real time. Second, in (Jobin et al., 2023), measurements were performed after 90 minutes of QPL incubation, while in the present study total observation time by TIRF microscopy was within 10 minutes of QPL incubation.

To address this apparent discrepancy, we measured with confocal microscopy the recruitment of βarr2-mCherry to intracellular FLAG-D2R clusters upon stimulation with QPL for 10 and 90 minutes (Figure S5). We observed intracellular FLAG-D2R clusters at both time points. Similar to results obtained after 30 minutes incubation (Figure 3), the number of clusters is significantly smaller in cells enriched in DHA or DPA (Figure S5B). However, the fluorescence of βarr2-mCherry at FLAG-D2R clusters is similar in all three conditions at 10 minutes incubation, while it is significantly higher in cells enriched in DHA at 90 minutes incubation. Therefore, the recruitment of βarr2-mCherry is differently modulated by the level of DHA and DPA, depending on the location (plasma membrane vs endosome) and the duration of QPL application. Thanks to these additional experiments, we can conclude that the results from the present study are not in contrast with what has been published before (Jobin et al., 2023).

Furthermore, a lot of additional mechanistic questions arise, which would need to be experimentally addressed to delineate what the mechanistic impact of altered PUFA levels is: What is the effect of DHA and DPA enrichment on G protein coupling to D2R? Is the GRK2 recruitment to and phosphorylation of D2R altered upon DHA and DPA enrichment? Are the receptor clusters co-localized with clathrin coated pits (CCPs)? And if so, why are receptor cluster unchanged if the link to the clathrin

coated machinery, i.e. beta-arrestin, is reduced? How do the changes in ligand affinity noted in the earlier work (ref 25, Fig. 1) relate to the findings of this study?

We agree with the reviewer that our work raises many questions regarding the molecular mechanisms at play in this effect. We have tried to address some of them. To get more insight into the possible mechanisms behind the observed modulation, we have measured the recruitment of an additional protein involved in D2R endocytosis, GIPC (Patel et al., 2024). Figure S6 shows that GIPC-mCherry recruitment to D2R clusters is minimal, compared to the robust recruitment of βarr2-mCherry, and not modulated by DHA or DPA treatments. Moreover, we have determined the recruitment of SEP-D2Rs to CCPs, visualized with CLC-mCherry (Figure S3). We show that SEP-D2Rs indeed cluster at CCPs and that this recruitment is not affect by treatments with DHA or DPA.

Figure 7 shows that internalization of the ICL2 serine double mutant of D2R is not affected by DHA and DPA enrichment, distinct to the wild-type receptor. While this is potentially interesting, the functionality of the mutated receptor needs to be tested. Do the mutations affect ligand affinity and G protein coupling? And see above comment on Fig. 4: is the serine motif, detected by the authors in other GPCRs, predictive for regulation by DHA and DPA?

We completely agree with the reviewer that a more comprehensive characterization is required to better interpret our results on mutant D2R. We have now measured QPL binding affinity to D2R mutant in cell membrane fragments using plasmon waveguide resonance (PWR) (Alves and Lecomte, 2019; Jobin et al., 2023). The data presented in Fig. S9 shows that QPL binds to the mutant receptor (S147,148A) with comparable affinity as to the WT receptor and that is modulated by PUFAs in the same way. The overall conformation signatures are also similar.

In sum, the finding of Sposini and colleagues that PUFAs impact D2R internalization and beta-arrestin recruitment are interesting. Yet several additional experiments outlined above are needed to make this a comprehensive study with new mechanistic insights.

We are pleased that the reviewer found our results generally interesting and we thank the reviewer for suggesting valuable additions to the manuscript.

Reviewer #2 (Remarks to the Author):

This interesting study uses live and fixed cell trafficking assays to demonstrate that incorporation of poly-unsaturated (PUFAs), but not saturated, free fatty acids into membrane phospholipids reduce agonist induced internalization of the D2 dopamine receptor. Pulsed pH live microscopy was used to demonstrate that the reduced internalization by incorporation of free fatty acid was accompanied by a blunted recruitment of Beta arrestin for the D2R. The manuscript sets out well-designed experiments and the observations are generally supported by their data. A particular strength is the validation of incorporation of FFA's in the plasma membrane. This builds on previous work looking at the modulation of D2R signalling and D2R-mediated behaviors by PUFAs. There does seem to be a discrepancy between the effect of PUFAs on arrestin recruitment between this study and the previous study (enhanced in the previous study, inhibited in this one) (Jobin et al. PMID: 37550407).

We thank the reviewer for their positive evaluation of our work. We address the apparent discrepancy with the previous study of some of the co-authors (Jobin et al., 2023) in our answer to the reviewer's query below.

The role of PUFAs in modulating D2R signalling has been studied before by the authors, with evidence to suggest that PUFAs modulate dopamine-controlled behaviors (Ducrocq et al. PMID: 32142670. and Jobin et al. PMID: 37550407). The present study does not extend to assess the physiological, pathophysiological or therapeutic role of the observations (except from referring the relation between FFAs and disease). The link between the findings of this study (reduced internalization and arrestin recruitment) and their previous work are not clear, particularly given the above discrepancy. The mechanism by which PUFAs might inhibit arrestin recruitment and subsequently internalization remains undefined although the authors provide some ideas in their discussion.

These are all fair points and, in considering them, we recognize a need to further clarify our experimental focus and rationale. Thus, we have revised the discussion part of our manuscript as well as re-conciliated the results from this work and a previous one (Jobin et al., 2023). Moreover, in the revised work, we have performed additional experiments to provide further mechanistic insight into the observed D2R signaling PUFA modulation. See below for experiments aimed at addressing specific points.

The authors show that the effect of the PUFAs to restricted to the D2R as compared to a battery of other selected GPCRs. They provide a rationale for this selective action and suggest a number of other receptors that might be sensitive to PUFAs. Confirming this hypothesis would broaden the interest and impact of the paper along with providing more mechanistic insight.

We agree with the reviewer that finding another GPCR which could be modulated by addition of DHA or DPA would extend the scope of our study. Unfortunately, we were unable to identify such a receptor. We have tried to assess the internalization of D3R with the DERET assay. However, as shown before (Kim et al., 2001), this receptor does not internalize, even with co-expression of GRK2 and βarrestin2. Therefore, we could not test the effect of PUFA enrichment. Regarding the other receptors, we could not get good expression levels in HEK-293 cells (NOP) or we did not have the constructs at hand (S1PR5, MTR1a). Nevertheless, we clearly show that the ligand-induced internalization of D2R is specifically sensitive of to the level of DHA or DPA in membranes.

Specific comments are as follows:

There appears to be a discrepancy between the authors' findings in this paper, namely that PUFAs inhibit D2R internalization and the recruitment of arrestin, and their previous study which illustrates that enrichment of PUFAs robustly increase agonist stimulated arrestin recruitment (Jobin et al. PMID: 37550407). The authors should explain this discrepancy.

We completely agree with this and the previous reviewer on the need to re-conciliate the apparent discrepancy between the present work and (Jobin et al., 2023) regarding β-arrestin recruitment and its modulation by PUFAs.

In the study by Jobin and co-workers, β-arrestin recruitment was assessed in CHO cells, using PathHunter assay with signal detection at 90 minutes after QPL addition., before and within 10 minutes of QPL stimulation. In addition to the fact that the experimental approaches used in the two studies

are different and measure slightly different properties, another important factor to is the time of stimulation with QPL. In previous experiments, measurements were performed after 90 minutes of QPL incubation, while total observation time by TIRF microscopy in the present study was within 10 minutes.

To address this apparent discrepancy, we measured with confocal microscopy the recruitment of βarr2-mCherry to intracellular FLAG-D2R clusters after incubations with QPL for 10 and 90 minutes (Figure S5). FLAG-D2R clusters were observed at both time points. Consistent with our results showing a reduced number of D2R endosomes at 30 minutes of incubation with QPL(Figure 3), the number of D2R clusters is significantly smaller in cells enriched in DHA or DPA at both 10 and 90 minutes of QPL stimulation(Figure S5B). Nonetheless, while the fluorescence of βarr2-mCherry at FLAG-D2R clusters is similar in all three conditions at 10 minutes incubation, it is significantly higher in cells enriched in DHA at 90 minutes incubation. Therefore, the recruitment of βarr2-mCherry is differently modulated by the level of DHA and DPA depending on the membrane (plasma membrane vs endosome) and the duration of QPL application. Thanks to these additional experiments, we can conclude that the results from the present study are not in contrast with what has been published before Jobin et al.). We have now added a specific sentence in the discussion to help readers re-conciliate the findings from the two papers.

The authors suggest that D2R internalization is dependent on GRK2 and B arrestin2. While there is certainly evidence of B arrestin 2 and GRK2 overexpression potentiating D2R internalization, the essential role of arrestin was not conclusively demonstrated with knock out/knock down of arrestin (https://doi.org/10.1074/jbc.M106728200). The recent work by Patel and colleagues (PMID: 39638791) demonstrates that, both in a HEK293 cell background and in striatal neurons, D2R internalization is independent of B arrestin but instead driven by the cytoskeletal motor myosin VI in a process that also involves a PDZ domain within the C-terminal tail of D2R, GIPC and is regulated by the intracellular loop 3 of the D2R. From that study it seems like that the level of D2R internalisation is unaffected by the knockout of B arrestin. Can the authors reconcile their findings with this study? Might PUFAs instead regulate this process? The authors could provide further insight regarding these two different mechanisms. The repetition of their findings in the CRISPR B arrestin K/O HEK cell lines would be a useful experiment (PMID: 29362459).

We thank the reviewer for these important remarks. We show in the revised manuscript that, similar to (Kim et al., 2001), overexpression of GRK2 and β-arrestin2 greatly increases D2R endocytosis after QPL application (Figure S1B). Moreover, we have addressed the role of GIPC by co-expressing GIPC-mCherry and measuring its recruitment to SEP-D2R clusters formed after application of QPL. Figure S6 shows that GIPC-mCherry recruitment to D2R clusters is minimal, compared to the robust recruitment of βarr2-mCherry, and not modulated by DHA or DPA treatments. We did not address the role of β-arrestins in KO cells because we may face compensation mechanisms. In all cases, we do not link the effect of PUFA enrichment to the specific interaction of D2R with a cytosolic effector protein. We simply note that the recruitment of β-arrestin2 to ligand-bound D2R is strongly modulated by the level of membrane PUFAs.

In the light of the discrepancy with their previous work regarding the effect of PUFAs on B arrestin recruitment, and the different mechanisms proposed to underlie agonist stimulated internalization of the D2R, the mechanistic gains from this study are unclear. It remains unclear exactly how PUFAs might modulate D2R internalisation and what how the effect on B arrestin recruitment to the D2R links to

receptor internalization. It is also not clear from their data what effect PUFAs have on D2R receptor function. From their previous study, Jobin et al. PMID: 37550407, enrichment of PUFAs has no effect on G protein signalling as measured in a cAMP and G protein activation assay. If arrestin recruitment and/or receptor internalization is inhibited/enhanced then we might expect some change in receptor G protein signalling?

We agree with the reviewer that our study raises a number of questions concerning D2R signaling in the conditions of DHA/DPA enrichment. To address this issue, we have performed pilot experiments assessing the role of DHA/DPA enrichment in ERK activation. We found in triplicate experiments illustrated in the Figure below that both DHA and DPA seem to reduce ERK phosphorylation induced by QPL application. However, the effects were variable and we did not have significant differences. Moreover, we noticed in cells treated with DPA a large increase in basal phosphorylated ERK, for which we have no explanation at the moment. Even if the reduced ERK1/2 phosphorylation shown in this figure is consistent with reduced Β arrestin recruitment at D2R clusters and endocytic vesicles, addressing the consequences of DHA and DPA on D2R signaling would require significant work which goes beyond the scope of this manuscript.

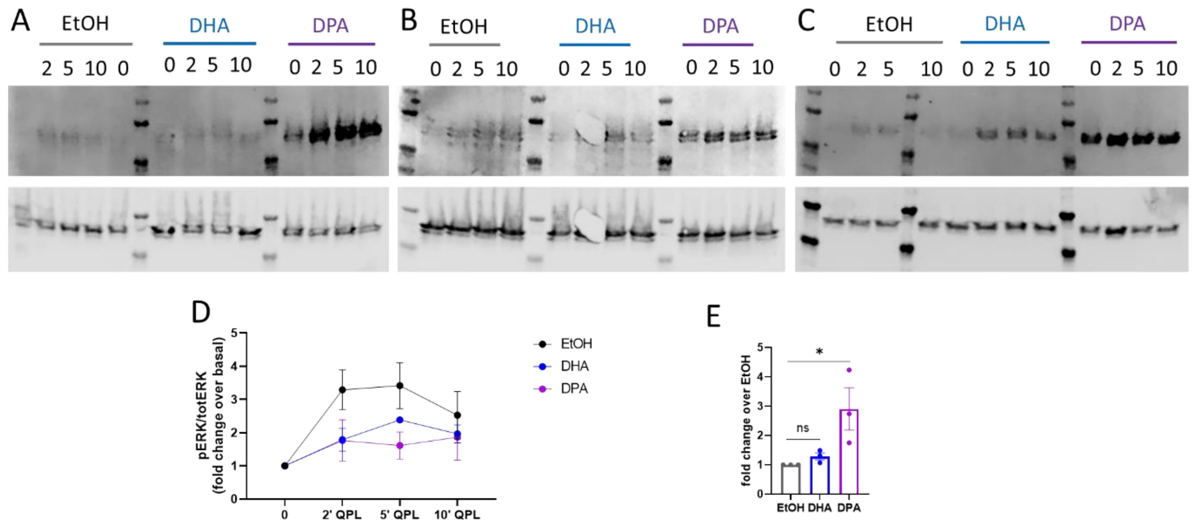

Figure: Effect of DHA and DPA treatment on phosphorylation of ERK1/2. Phosphorylation of ERK 1/2 was determined by western blot at stated time points after QPL stimulation in HEK293 cells expressing FLAG-D2R, βARR2 and GRK2. Cells were serum starved for 6 hours prior to stimulation with QPL. Total ERK was used as a loading control. **A-C**, Blots from three independent experiments. Top: phospho ERK1/2; Bottom: total ERK1/2. For the detailed Western blot protocol please see (Sposini et al., 2017). **D**, Densitometry analysis of ERK 1/2 phosphorylation normalized to the 0-min time point for each condition. n = 3 independent experiments shown in A-C. **E**, Basal levels of ERK 1/2 phosphorylation in the three different conditions calculated from the blots shown in A-C. N=3 independent experiments; Ordinary one-way ANOVA followed by Dunnett's multiple comparison test; * $p<0.05$ (exact p-values: EtOH vs DHA = 0.8484, EtOH vs DPA = 0.0329).

The use of the mutant receptor to ablate PUFA sensitivity of the receptor is nice but what exactly these residues do is not clear. The authors propose that enriching PUFAs will cause ICL2 to be buried in the membrane but it is not clear what effect this will then have. Which cytosolic proteins will no longer interact with the D2R and why? Are these serines phosphorylated? Do you see reduced phosphorylation of these residues in the presence of PUFAs? Do PUFAs impact the recruitment of GRKs? Can the authors measure how PUFA changes its interaction with GIPC? It is a nice observation that relatively few GPCRs have the double serines in ICL2 and it would certainly add to the study to

demonstrate that some of the others that have this motif are also sensitive to PUFAs. If you add this motif to a non-PUFA sensitive GPCR, do you confer sensitivity? This would provide more evidence of the proposed mechanism.

These are all excellent points that we have not yet addressed. Our goal in the present work is to establish the effect of DHA and DPA on D2R trafficking, specifically endocytosis. We believe the new data included in the revised manuscript help better defining the mechanistic insights of PUFAs regulation of D2R endocytosis, for example by including data on D2R clustering at CCPs (Figure S3) and on GIPC recruitment (Figure S6). However, some of the work proposed goes beyond the current paper objectives. We have included some of the reviewer's valuable ideas in the revised Discussion section.

There is a question of how the insights gained in this study in a HEK293 cell background will translate into a disease-relevant cell background. Is there not the possibility that such observations will be cell dependent? Could the authors validate their findings in, for example, striatal neuronal cultures to demonstrate the same impact of PUFAs on D2R internalization.

We thank the reviewer for bringing up this important issue. We have performed internalization experiments of FLAG-D2R in rat primary cortical neurons (which endogenously express D2Rs). In these cells, addition of QPL induces the robust internalization of D2R. Importantly, we show in Figure 5E,F that treatment with DHA or DPA decreases D2R internalization, consistent with the results obtained with HEK-293 cells.

Why did the authors choose to overexpress both GRK2 and B2 arrestin in this study and what impact this has on their observations? Did they try analogous experiments without this over expression?

This is a good point and important to clarify. We now show in Figure S1B that in HEK-293 cells transfected with SNAP-D2R alone, addition of the D2R agonist quinpirole (QPL, 10 µM) increases its internalization less than 2-fold (compared to cells without agonist). Co-transfection of cells with GRK2 (which phosphorylates D2R) and/or βarrestin2 (which binds phosphorylated D2R), increases receptor internalization 4 to 6 fold. This gives a much clearer assay to determine the effect of enrichment with fatty acids. Therefore, we used throughout this study HEK-293 co-transfected with tagged D2R, GRK2 and βarrestin2 at 1/1/1 ratio. To address the second point, we have performed internalization experiments of FLAG-D2R in rat primary cortical neurons. In these cells, addition of QPL induces the robust internalization of D2R. Importantly, we show in Figure 5E,F that treatment with DHA or DPA decreases D2R internalization, consistent with the results obtained with HEK-293 cells.

D2R internalization is measured over an 80-minute timescale, and significant internalisation appears to take about 30 minutes. How does this relate to the relatively transient release and uptake of dopamine at a synapse?

This is an interesting idea. We used 30 minutes of application of D2R agonists to measure internalization in fixed cells (Figure 3, 4 and 8). However, with live cell imaging, we detect internalization in the first DERET measures after application of agonist (every 5 minutes in Figure 4 and 8). In Figure 2, which corresponds to earlier experiments, the kinetics of internalization measured with DERET are slower, likely due to a different way of mixing solutions. Finally, in the single cell TIRF imaging of endocytosis using the ppH assay shown in Figure 7, we detect endocytic vesicles (assayed

with time lapse imaging every 4 s) within the first minutes of QPL application. In all live imaging assays, the effect of PUFA treatments on D2R endocytosis are visible at the earliest time points of our measures. Therefore, we propose that alterations in FA content affects D2R internalization, which could occur even after transient release of dopamine in brain tissue. Moreover, it should be noted that basal brain dopamine levels might fluctuate on a longer time scale, which could condition the level of D2R endocytosis in a time scale of minutes or more.

In their analysis of microscopy it appears that the authors combine the data from 3 independent experiments (of x number of cells per condition) together before analysing with ANOVA. With all data points included together it would seem there is the possibility that data from one experimental repeat might unduly influence this analysis. Why weren't mean effects of each experimental repeat taken and then these combined (so three mean values) and compared across the different conditions?

We show in the figure below the graphs corresponding to the analysis requested by the reviewer of the data presented in Figure 3 of our study (original panels B and D). We have separated the cells analyzed in the three individual experiments and we show the average quantification for each experiment in panels B' and D'. These two examples clearly show that the results reported by measures in individual cells (panels B and D) are not biased by single experiments. Moreover, we think that the representation of averages of experimental sessions does not fully represent the variance of measures, and any other representation would be more confusing than useful. Therefore, we would like to keep our representation of individual cells, as it is the norm in most current studies. Nevertheless, in the source data, which we will release upon manuscript acceptance, we will mark measures coming from individual experimental sessions.

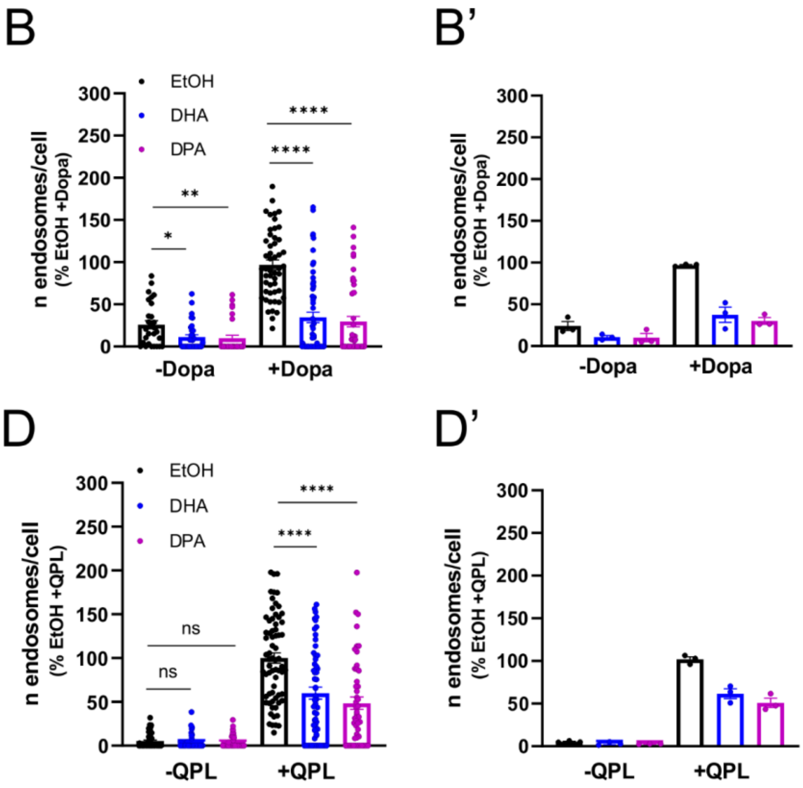

Figure: B and D represent the panels B and D from Figure 3 in the present manuscript. B': same data as in B with averages of 9-25 cells per experimental session. D': same data as in D with averages of 10-27 cells per experimental session. Bars and error bars represent mean ± SEM of 3 individual experiments.

Reviewer #3 (Remarks to the Author):

Over the past year, the authors have substantially revised the manuscript and thoroughly addressed my previous concerns regarding the interpretation of their findings. In the earlier version, the narrative could be interpreted as suggesting that blunted D2R internalization reflected cellular dysfunction, implying that increased PUFA levels negatively impact cellular physiology. In the current version, however, the authors have clarified that the observed reduction in D2R internalization is not inherently beneficial or detrimental, but rather a direct consequence of altered membrane PUFA content. This neutral, mechanistic framing is well-supported by the data.

We warmly thank the reviewer for their positive comments on our study and its improvement. We hope that the current revision has further increased the quality of our manuscript.

Only a few minor points remain that should be addressed or clarified before the manuscript is suitable for publication:

1. I appreciate the inclusion of Figure 1 in the main text and recognize the effort to clarify the effects of DHA, DPA, and BA on fatty acid composition. It is clear that DHA and DPA increase the proportion of polyunsaturated fatty acids (PUFAs) while reducing saturated fatty acids (SFAs), whereas BA increases SFAs with minimal impact on PUFA levels. However, the data remain somewhat puzzling in the context of mass conservation. If total fatty acids (Total FA) are composed of PUFAs and SFAs, an inverse relationship between these two categories would be expected. Yet this relationship is not apparent in Figure 1C. Additionally, the combined percentages from Figures 1B and 1C do not appear to sum to 100%. For instance, DHA treatment shows approximately 15% PUFA in Figure 1B and 35% PUFA in Figure 1C - totaling only 50%, which is unexpectedly low. Does this imply that monounsaturated fatty acids (MUFAs) constitute a substantial portion of the Total FA and are not explicitly represented in these panels? Clarifying this apparent discrepancy and the role of MUFAs in the overall fatty acid balance is necessary for accurate interpretation of the data.

Indeed, the reviewer is right in considering that the rest of FAs in extracted phospholipids is composed of monounsaturated FAs. The graph below represents the proportion of MUFAs in HEK-293 cells after treatment, which varies to accommodate the changes in PUFA levels :

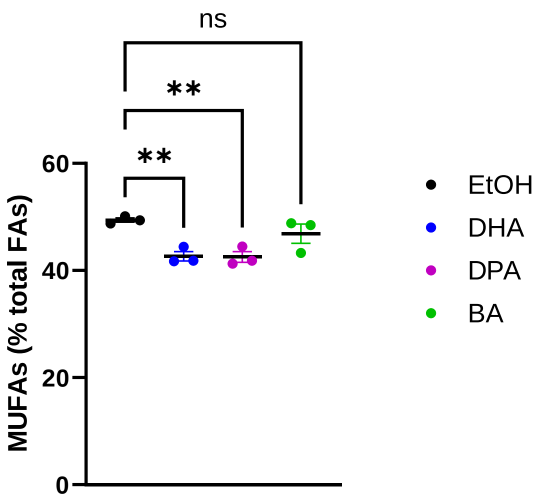

To clarify this point, we have added in the Methods section: "Fatty acid methyl esters were identified by comparison with commercial and synthetic standards of saturated, monounsaturated and polyunsaturated FAs (see list in source data) and data analysis were processed using the Galaxie software (Varian)."

2. Figures 1D-F clearly show that incubation with DHA, DPA, and BA results in an increased abundance of the respective fatty acids in the lipidomic analysis. However, it remains unclear whether these fatty acids are truly metabolized and incorporated into cellular membranes - particularly the plasma membrane - or if they are simply retained within the cell as free fatty acids, stored in lipid droplets, or peripherally associated with membranes in a way that allows them to be extracted during sample preparation. Additional clarification is needed in the manuscript to distinguish between these possibilities and to support the conclusion that the observed lipidomic changes reflect bona fide membrane incorporation.

Incubation of cells with free fatty acids – especially PUFAs - has been largely shown to lead to efficient incorporation into phospholipids, particularly in phosphatidylethanolamines (PEs) and Phosphatidylcholines (PCs) (Goustard-Langelier et al., 2000; Kim et al., 2000). In many models, the majority of excess exogenous free fatty acids that is not immediately oxidized is esterified into neutral lipids and deposited in lipid droplets over hours of incubation (Obaseki et al., 2024). In addition, some of us recently reported through Raman imaging studies on PUFA-enriched HEK-293 cells that such PUFAs are found at the plasma membrane (Baccouch et al., 2023). Therefore, it is very likely that the effects reported directly result from plasma membrane enrichment in PUFA-containing phospholipids.

References

Alves ID, Lecomte S (2019) Study of G-Protein Coupled Receptor Signaling in Membrane Environment by Plasmon Waveguide Resonance. Acc Chem Res 52:1059–1067.

Baccouch R, Shi Y, Vernay E, Mathelié-Guinlet M, Taib-Maamar N, Villette S, Feuillie C, Rascol E, Nuss P, Lecomte S, Molinari M, Staneva G, Alves ID (2023) The impact of lipid polyunsaturation on the physical and mechanical properties of lipid membranes. Biochimica et Biophysica Acta (BBA) - Biomembranes 1865:184084.

Bazinet RP, Layé S (2014) Polyunsaturated fatty acids and their metabolites in brain function and disease. Nat Rev Neurosci 15:771–785.

Goustard-Langelier B, Alessandri J m., Raguenez G, Durand G, Courtois Y (2000) Phospholipid incorporation and metabolic conversion of n-3 polyunsaturated fatty acids in the Y79 retinoblastoma cell line. Journal of Neuroscience Research 60:678–685.

Jobin M-L et al. (2023) Impact of membrane lipid polyunsaturation on dopamine D2 receptor ligand binding and signaling. Mol Psychiatry 28:1960–1969.

Kim H-Y, Akbar M, Lau A, Edsall L (2000) Inhibition of Neuronal Apoptosis by Docosahexaenoic Acid (22:6n-3): ROLE OF PHOSPHATIDYLSERINE IN ANTIAPOPTOTIC EFFECT*. Journal of Biological Chemistry 275:35215–35223.

Kim KM, Valenzano KJ, Robinson SR, Yao WD, Barak LS, Caron MG (2001) Differential regulation of the dopamine D2 and D3 receptors by G protein-coupled receptor kinases and beta-arrestins. J Biol Chem 276:37409–37414.

Obaseki E, Adebayo D, Bandyopadhyay S, Hariri H (2024) Lipid droplets and fatty acid-induced lipotoxicity: in a nutshell. FEBS Letters 598:1207–1214.

Patel NM, Ripoll L, Peach CJ, Ma N, Blythe EE, Vaidehi N, Bunnett NW, von Zastrow M, Sivaramakrishnan S (2024) Myosin VI drives arrestin-independent internalization and signaling of GPCRs. Nat Commun 15:10636.
Sposini S, Jean-Alphonse FG, Ayoub MA, Oqua A, West C, Lavery S, Brosens JJ, Reiter E, Hanyaloglu AC (2017) Integration of GPCR Signaling and Sorting from Very Early Endosomes via Opposing APPL1 Mechanisms. Cell Reports 21:2855–2867.

REVIEWER COMMENTS

We have reproduced the reviewers' queries in black and written our answers in blue. We have attached the complete list of references used in these answers at the end of this document.

Reviewer #1 (Remarks to the Author):

The revised manuscript by Sposini and colleagues contains several new datasets that strengthen the conclusion that PUFA levels affect agonist-driven D2R internalization (in GRK and beta-arrestin overexpression conditions, see below). Also, the rationale for using the specific PUFA enrichment protocol is now clearer (revised Fig. 1), and the new results showing reduced D2R internalization upon increasing PUFA levels in cultured neurons represent a valuable addition (Fig. 5).

We thank the reviewer for their positive assessment of our revised manuscript. We address the five remaining points.

Based on my previous comments and the newly added data, the following points remain:
1. The authors now show that β-arrestin and GRK2 overexpression strongly enhances agonist-driven D2R internalization, which they use as the rationale for employing these conditions throughout the study (Fig. S1). However, given that agonist-driven D2R internalization is still detectable in cells not overexpressing these regulators (2-fold over basal), and considering previous work establishing that D2R internalization occurs independently of β-arrestin (Patel et al., 2024), it is important to test whether PUFA levels affect D2R internalization in cells not overexpressing GRK2 and β-arrestin. This straight-forward experiment is both meaningful and important to for the broader validity of the findings.

We have now performed the experiment requested by the reviewer, presented in Figure 3E,F. We show with anti-FLAG internalization and confocal microscopy that, in HEK293 cells transfected with FLAG-D2R only, the number of internalized clusters (endosomes) is reduced by half in cells treated with DHA or DPA, just like in HEK293 cells overexpressing FLAG-D2R, GRK2 and β-arrestin2 (Figure 3C,D). We write in the Results: "Moreover, in HEK293 transfected only with FLAG-D2R, application of QPL induces significant receptor endocytosis that is also significantly reduced in cells treated with DHA or DPA (61.8 ± 6.9 % of control for DHA and 48.5 ± 7.3 % DPA, respectively, $p < 0.0001$)(Figure 3E,F)." Therefore, overexpression of GRK2 and β-arrestin2, while enhancing D2R internalization, does not alter PUFA impact in this pathway.

2. The hypothesis that the two-serine motif in the ICL2 of GPCRs contributes to PUFA-regulated internalization was not tested for other GPCRs. Plasmids for GPCR constructs are readily available (e.g., via Addgene), making it surprising that this was not investigated, particularly given that two reviewers raised this point.

As written in our previous answer to the reviewers' comments, we have tried to measure internalization of D3R and nociceptin receptor (NOP). We show here the results obtained with the DERET assay. For the D3R, we measure expression of D3R at the plasma membrane but very little internalization (Figure A1), as shown before (Kim et al., 2001).

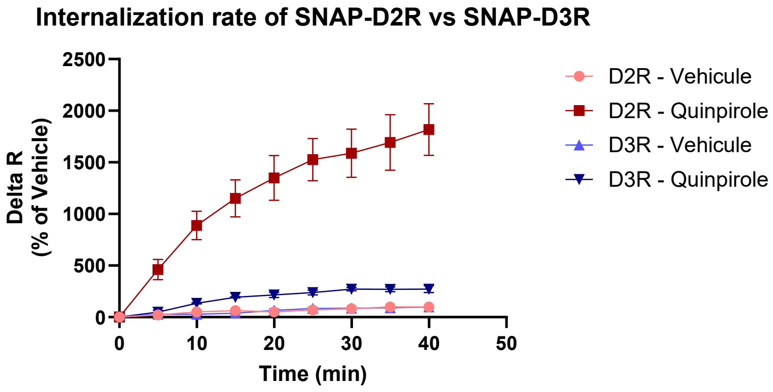

Figure A1: SNAP-D3R shows little endocytosis by the DERET assay. Comparison of the internalization of D2R and D3R induced by quipirole (10 µM). In cells co-transfected with SNAP-D3R, GRK2 and β-arrestin2, the increase is minimal, precluding further analysis on the effect of pretreatment with DHA and DPA. Each point represents an average of 3 independent experiments.

For the NOP (Figure A2), we could not get good expression at the plasma membrane. There are only very few reports of expression of NOP and internalization in HEK293 cells (Corbani et al., 2004) which have not been followed up since then. We also note that, in a recent article on the development of a nociceptin fluorescent reporter based on NOP, the authors had to modify WT NOP by exchanging the C-terminal tail with the one of the kappa opioid receptor to get good expression at the plasma membrane (Zhou et al., 2024). Therefore, despite our attempts, we could not get any conclusive data on the sensitivity of GPCRs other than D2R to the PUFA composition of cellular membranes. This point could be addressed in further studies.

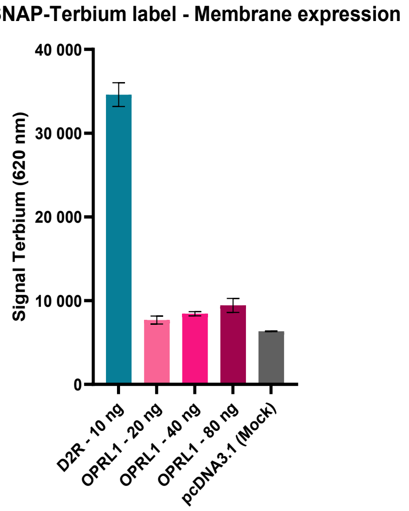

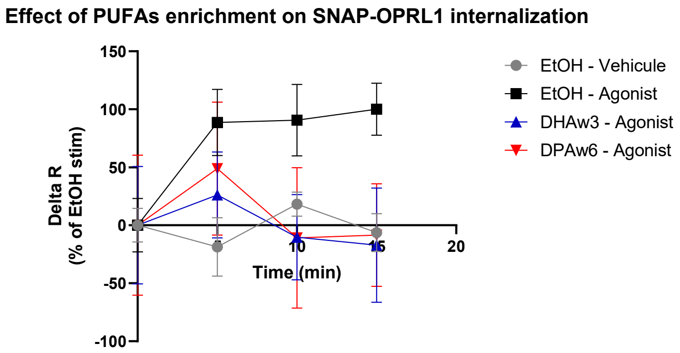

Figure A2: Low expression of SNAP-NOP at the plasma membrane leads to inconclusive effects of DHA or DPA treatments on its internalization. Left, labelling of SNAP-D2R or SNAP-NOP with SNAP-Terbium in various conditions of cell transfection. While SNAP-D2R had good expression leading to a large signal enabling the confirmation of the effect of DHA or DPA treatment on D2R internalization (not shown for this dataset, similar to Figure 2A and 4F), SNAP-OPRL1 (=SNAP-NOP) expression remains very low even with increased amount of DNA, from our standard 20 ng, to 80 ng. Consequently, the effect of DHA or DPA treatment on SNAP-OPRL1 endocytosis induced by 1 µM nociception is inconclusive.

3. The new data on GIPC recruitment do not represent a reliable or conclusive addition to the manuscript (Fig. S6), and I recommend removing this section for the following reasons: First, GIPC

recruitment was assessed in cells overexpressing GRK and β-arrestin, which are likely to sterically compete with GIPC binding, especially given their overexpression. Second, no convincing agonist-driven GIPC recruitment above background was detected (Fig. S6E), making it impossible to conclude that GIPC recruitment is unaffected by changes in PUFA levels, as stated on p. 13.

We have tested the possibility that GIPC competes with β-arrestin in cells expressing SEP-D2R and GIPC-mCherry only. As shown in the figure below, GIPC-mCherry is, like in cells expressing SEP-D2R, GIPC-mCherry, GRK2 and β-arrestin2, not detected in clusters of SEP-D2R after application of the D2R agonist quinpirole (10 µM).

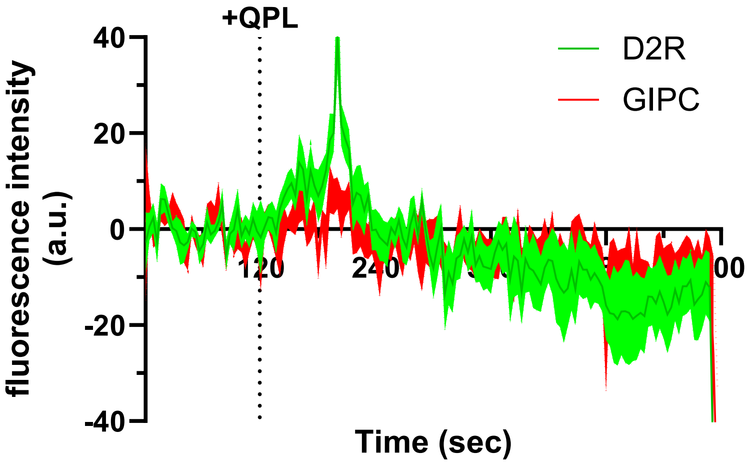

Figure A3: Average fluorescence within each SEP-D2R cluster minus average fluorescence before agonist addition (calculated as in Figure 6B,E of the manuscript) for 7 cells transfected with SEP-D2R and GIPC-mCherry. In these conditions, the clustering of SEP-D2R after QPL was scarce, unlike in cells transfected with SEP-D2R, GRK2 and β-arrestin2-mCherry (Figure 6 and Figure 7 of the manuscript).

We agree with the reviewer that this section "do[es] not represent a reliable or conclusive addition to the manuscript", so we have removed it, along with the corresponding text in the Results, Methods and Discussion.

4. I appreciate that the authors attempted to address discrepancies between the present study and their previous work by examining β-arrestin recruitment to D2R clusters at longer time points following PUFA manipulation. However, β-arrestin colocalization with D2R clusters at 90 minutes is not evident based on the images provided (neither EtOH control, nor DHA and DPA conditions) (Fig. S5). It would be important to show the individual β-arrestin and D2R images in grayscale, in addition to the merged images, to allow a clearer assessment of the validity of these data.

We now provide the individual images in grayscale in Figure S5. Some punctuate staining of β-arrestin-mCherry can be seen, albeit not systematically colocalized with FLAG-D2R.

5. In the discussion, I find this following paragraph problematic and strongly recommend rewording or deletion:
"However, activation of β-arrestin by GPCRs can occur through a recently described catalytic pathway which does not require the formation of a stable complex (ref 43,62). In this context, transient activation of membrane-associated β-arrestin by interaction with GPCRs drives its association with CCPs (ref 62). It is thus possible to decouple D2R recruitment from β-arrestin recruitment to CCPs, resulting in DHA or DPA membrane enrichment only affecting D2R endocytosis and not its clustering

at CCPs." The cited studies show that clustering of β-arrestin in CCPs can occur independently of GPCR recruitment to CCPs, however, they do not demonstrate that GPCR clustering in CCPs is decoupled from β-arrestin. This distinction is important, as the authors use these citations to support the possibility that D2R clustering in CCPs is decoupled from β-arrestin, which is not justified by the referenced work.

In this part of the Discussion, we propose a mechanism to explain our observation that enrichment of cell membranes with DHA or DPA reduces D2R endocytosis and β-arrestin recruitment to CCPs without altering D2R recruitment to CCPs. References 45 (Eichel et al., 2018) and 64 (Grimes et al., 2023) show possible uncoupling between GPCR and β-arrestin recruitment to CCPs. We have expanded this paragraph to make it clearer:

"However, activation of β-arrestin by GPCRs can occur through a recently described catalytic pathway which does not require the formation of a stable complex (Eichel et al., 2018; Grimes et al., 2023). In this context, recruitment of β-arrestin2 can occur without recruitment of the stimulated GPCR (β1AR) to CCPs (Eichel et al., 2018). Conversely, recruitment of stimulated β2AR still occurs without recruitment of mutated β-arrestin2 (Eichel et al., 2018). Moreover, single particle imaging revealed that transient activation of membrane-associated β-arrestin by interaction with GPCRs can drive its association with CCPs without stable association (Grimes et al., 2023). It is thus possible to decouple D2R recruitment from β-arrestin recruitment to CCPs, resulting in DHA or DPA membrane enrichment only affecting D2R endocytosis and not its clustering at CCPs."

Therefore, we would like to keep this part of the Discussion because this is for us the only way to interpret our results concerning recruitment to CCPs prior to receptor endocytosis.

Reviewer #2 (Remarks to the Author):

In their revised manuscript, Sposini and colleagues have added a significant amount of additional data and discussion to address almost all of my comments. I have only minor comments remaining.

They have provided additional data and explanation around the discrepancy between the findings of this paper and that of Jobin et al. in terms of the effect of PUFAs on arrestin recruitment to the D2R. I think it would be useful for the reader to have a bit more detail explaining explicitly the nature of the previous experiment (whole cell assay of arrestin recruitment that does not distinguish between the D2R at the PM and at intracellular compartments) in addition to the difference in agonist incubation time. I think the authors are implying that the previous experiment in Jobin et al. is is likely that the measurment is of arrestin recruitment to D2Rs within the endosome. If so I think this is worth stating explicitly.

We thank the reviewer for this suggestion. We have now added in the Results section, before the presentation of the experiment illustrated in Figure S5 (page 8):

"In this assay [in ref 21], the measured βarr2 recruitment could occur in endosomes, where most receptors reside after prolonged activation, rather than on the plasma membrane, as we measured here with TIRF microscopy."

From a mechanistic point of view can the authors speculate why after 90 minutes they do see an increase in arrestin recruitment to the D2R in intracellular compartments with the addition of PUFAs, what changes occur to reverse the initial effect of decreasing arrestin recruitment which is presumably occurring predominantly at the PM?

We propose that these differences may arise from the different lipid composition of plasma membrane at the initial phase of agonist stimulation and endosomes (in which most D2R is located after 90 minutes of stimulation). We have added in the Results:

"Therefore, the recruitment of βarr2-mCherry is differently modulated by the level of DHA and DPA depending on the location (plasma membrane *vs* endosome, which may have a different lipid composition [46]) and the duration of QPL application."

The addition of data for the internalisation of Flag-D2R in cortical primary neurons is a nice addition and shows that the effects in these cells match those seen in their HEK experiments. The additional data around clustering at CCPs and the experiments looking at GIPC recruitment are also useful additions.

We thank the reviewer for their comments. However, to comply with reviewer #1's suggestion, we have now removed the experiments looking at GIPC recruitment.

There are some additional questions that the authors were not able to address but I agree that such questions, such as the impact of PUFAs on D2R signalling lie beyond the scope of this manuscript.

We thank the reviewer for acknowledging the need for our study to remain focused on the effect of PUFA levels on D2R endocytic trafficking.

Reviewer #3 (Remarks to the Author):

The authors have thoroughly addressed my first comment regarding mass balance on the lipid tails directly in the revised manuscript.

With respect to my second concern about interpreting the lipidomic shifts after DHA/DPA/BA incubation, the authors provided a thorough and well-reasoned explanation in their response, including relevant literature. However, this clarification is not reflected in the manuscript. As written, the manuscript infers membrane enrichment from whole-cell phospholipid measurements without explicitly addressing whether these changes reflect plasma membrane incorporation versus other cellular lipid pools. This clarification is important for the strength of the mechanistic interpretation and should be incorporated into the manuscript. I therefore ask that the authors incorporate a brief version of their explanation into the manuscript (Results and/or Discussion), with appropriate citations and an explicit statement of the assumptions regarding plasma membrane enrichment.

We thank the reviewer for this suggestion. In the first part of the Results section, we have added references 36 (Kim et al., 2000) and 37 (Goustard-Langelier et al., 2000) as well as the following sentence:

"In addition to these measures of total FAs, we have shown before with Raman imaging of PUFA-enriched HEK293 cells that most added PUFAs are incorporated in the plasma membrane (Baccouch et al., 2023)."

References:

Baccouch R, Shi Y, Vernay E, Mathelié-Guinlet M, Taib-Maamar N, Villette S, Feuillie C, Rascol E, Nuss P, Lecomte S, Molinari M, Staneva G, Alves ID (2023) The impact of lipid polyunsaturation on the physical and mechanical properties of lipid membranes. Biochimica et Biophysica Acta (BBA) - Biomembranes 1865:184084.

Corbani M, Gonindard C, Meunier J-C (2004) Ligand-Regulated Internalization of the Opioid Receptor-Like 1: A Confocal Study. Endocrinology 145:2876–2885.

Eichel K, Jullié D, Barsi-Rhyne B, Latorraca NR, Masureel M, Sibarita J-B, Dror RO, von Zastrow M (2018) Catalytic activation of β-arrestin by GPCRs. Nature 557:381–386.

Goustard-Langelier B, Alessandri J m., Raguenez G, Durand G, Courtois Y (2000) Phospholipid incorporation and metabolic conversion of n-3 polyunsaturated fatty acids in the Y79 retinoblastoma cell line. Journal of Neuroscience Research 60:678–685.

Grimes J, Koszegi Z, Lanoiselée Y, Miljus T, O'Brien SL, Stepniewski TM, Medel-Lacruz B, Baidya M, Makarova M, Mistry R, Goulding J, Drube J, Hoffmann C, Owen DM, Shukla AK, Selent J, Hill SJ, Calebiro D (2023) Plasma membrane preassociation drives β-arrestin coupling to receptors and activation. Cell 186:2238-2255.e20.

Kim H-Y, Akbar M, Lau A, Edsall L (2000) Inhibition of Neuronal Apoptosis by Docosahexaenoic Acid (22:6n-3): ROLE OF PHOSPHATIDYLSERINE IN ANTIAPOPTOTIC EFFECT *. Journal of Biological Chemistry 275:35215–35223.

Kim KM, Valenzano KJ, Robinson SR, Yao WD, Barak LS, Caron MG (2001) Differential regulation of the dopamine D2 and D3 receptors by G protein-coupled receptor kinases and beta-arrestins. J Biol Chem 276:37409–37414.

Zhou X et al. (2024) Development of a genetically encoded sensor for probing endogenous nociceptin opioid peptide release. Nat Commun 15:5353.
